# Islet-Like Structures Generated In Vitro from Adult Human Liver Stem Cells Revert Hyperglycemia in Diabetic SCID Mice

**DOI:** 10.1007/s12015-018-9845-6

**Published:** 2018-09-06

**Authors:** Victor Navarro-Tableros, Chiara Gai, Yonathan Gomez, Sara Giunti, Chiara Pasquino, Maria Chiara Deregibus, Marta Tapparo, Adriana Pitino, Ciro Tetta, Maria Felice Brizzi, Camillo Ricordi, Giovanni Camussi

**Affiliations:** 1 2i3T – Scarl.-Molecular Biotechnology Center (MBC), University of Turin, Via Nizza, 52, 10126 Turin, Italy; 2 Department of Medical Sciences, University of Turin, Corso Dogliotti 14, 10126 Turin, Italy; 3 Fondazione per la Ricerca Biomedica-ONLUS, Via Nizza, 52, 10126 Turin, Italy; 4 Molecular Biotechnology and Health Sciences, MBC, Via Nizza, 52, 10126 Turin, Italy; 5 Unicyte AG, Oberdorf, Switzerland; 6 Diabetes Research Institute, University of Miami, Miami, FL USA

**Keywords:** Pancreatic islets, Pancreatic β cells, Insulin-producing stem cells, Diabetes, Liver stem cells, 3D culture

## Abstract

A potential therapeutic strategy for diabetes is the transplantation of induced-insulin secreting cells. Based on the common embryonic origin of liver and pancreas, we studied the potential of adult human liver stem-like cells (HLSC) to generate in vitro insulin-producing 3D spheroid structures (HLSC-ILS). HLSC-ILS were generated by a one-step protocol based on charge dependent aggregation of HLSC induced by protamine. 3D aggregation promoted the spontaneous differentiation into cells expressing insulin and several key markers of pancreatic β cells. HLSC-ILS showed endocrine granules similar to those seen in human β cells. In static and dynamic in vitro conditions, such structures produced C-peptide after stimulation with high glucose. HLSC-ILS significantly reduced hyperglycemia and restored a normo-glycemic profile when implanted in streptozotocin-diabetic SCID mice. Diabetic mice expressed human C-peptide and very low or undetectable levels of murine C-peptide. Hyperglycemia and a diabetic profile were restored after HLSC-ISL explant. The gene expression profile of in vitro generated HLSC-ILS showed a differentiation from HLSC profile and an endocrine commitment with the enhanced expression of several markers of β cell differentiation. The comparative analysis of gene expression profiles after 2 and 4 weeks of in vivo implantation showed a further β-cell differentiation, with a genetic profile still immature but closer to that of human islets. In conclusion, protamine-induced spheroid aggregation of HLSC triggers a spontaneous differentiation to an endocrine phenotype. Although the in vitro differentiated HLSC-ILS were immature, they responded to high glucose with insulin secretion and in vivo reversed hyperglycemia in diabetic SCID mice.

**Electronic supplementary material:**

The online version of this article (10.1007/s12015-018-9845-6) contains supplementary material, which is available to authorized users.

## Introduction

Type 1 diabetes (T1DM) is a disease with an important socio-economic impact, caused by autoimmune destruction of pancreatic insulin producing cells (β-cells) and associated with development of debilitating macrovascular and microvascular complications [1]. Treatment with exogenous insulin to attain a glycemic control does not completely prevent long term complications. Both pancreas and islets transplantation restore islets function and potentially reduce long-term diabetic complications [2, 3], but are limited by both donor shortage and need for immunosuppression. β cell replacement is an attractive therapy to achieve normoglycemia in patients with diabetes mellitus and several studies focused on generation of human β cells [4]. Pluripotent stem cells for their unlimited capacity of self-renewal may represent a scalable source of insulin producing cells [5–17]. β-like cells have been differentiated from human induced pluripotent stem cells (hiPCS) [18–20] and human embryonic stem cells (hESC) [13, 21]. Nevertheless, most current approaches are complex and require virus-induced transfection or methods of genetic engineering [4]. The challenge of producing β cells from pluripotent stem cells faces several inefficiencies including: rate of differentiation, generation of immature insulin producing cells, ethical concern, tumors formation or failure to maintain a sustained correction of hyperglycemia [5–17]. Recently, many efforts have been focused on identifying alternative and abundant cell sources of functional β cells [18–22]. In nature, pancreatic islets contain different endocrine cells: α, β, γ, ε and PP cells that secrete glucagon, insulin, somatostatin, ghrelin and pancreatic polypeptide. The characteristic conformation of pancreatic islets is important for a fine regulation of the islet function. In particular, it has been demonstrated that paracrine cell interactions among the insular cells help to modulate the hormone secretion [23, 24] and the tuning of normal β cell function [25]. Several multistep protocols have been described [6, 17, 26–33] to differentiate human embryonic or inducible pluripotent stem cells into pancreatic progenitors able to undergo in vivo maturation with generation of glucose sensitive insulin secreting cells able to reverse diabetes in mice [17, 28, 34]. The generation of in vitro mature endocrine cells would advantage reversal of diabetes. However, progenitors generated in vitro are immature and the protocols mainly produce poly-hormonal cells concomitantly expressing insulin, glucagon and somatostatin [12, 14, 27, 29, 30]. Rezania et al. have recently described a seven-stage protocol that generate glucose responsive insulin-secreting cells from human embryonic stem cells. These cells, even if not fully mature, were able to rapidly reverse diabetes [28].

Insulin producing cells may also be generated from pancreatic progenitors [27]. Despite an immature phenotype with poly-hormonal expression, the cells are able to revert or prevent diabetes in mice [28, 35]. Insulin producing cells have been obtained also by trans-differentiation of adult stem cells, for instance islet-derived mesenchymal stem cells cultured in absence of serum and with an appropriate mix of factors [36], as well as bone marrow mesenchymal stem cells in conditions mimicking diabetic niche [37]. Other approaches focused on reprogramming pancreatic cell types such as ductal, acinar or alpha cells by inducing epigenetic changes [38–40].

Both liver and pancreas share common embryonic origins and the developing liver has been shown to exhibit transcriptional features similar to the adult pancreas [41, 42]. Insulin producing cells were also generated by reprogramming hepatocytes inducing overexpression of PDX1 [43–46] plus exendin-4 to force proliferation and differentiation [47]. However, a limitation in using human liver hepatocytes is their low proliferative potential in vitro. Herrera et al. isolated from the adult human liver by a culture strategy leading death of mature hepatocyte a cell population with high proliferative capacity defined as adult human liver stem-like cells (HLSC). HLSC are clonogenic and express markers of both immature hepatocytes and mesenchymal stem cells [48]. Moreover, HLSC express several stem cells and embryonic markers, such as SSEA4, Oct4, SOX2 and nanog [48, 49] and are able to undergo multiple differentiations including islet-like phenotype [48]. However, the differentiation protocol was limited by a very low efficiency and the insulin secretion under glucose control was not demonstrated [48].

The aim of the present study was to investigate: 1. the potential of HLSC to generate in vitro insulin-producing 3D spheroid structures by a one-step protocol based on charge-dependent aggregation; 2. whether spheroid formation may allow endocrine differentiation of HLSC into islet-like structures (HLSC-ILS); 3. whether HLSC-ILS may secrete human C-peptide in vitro in response to glucose and reduce hyperglycemia in vivo in streptozotocin-diabetic SCID mice.

## Methods

### Isolation, Characterization and Culture of Human Liver Stem-Like Cells (HLSC)

HLSC were generated starting from cryopreserved human normal hepatocytes purchased from Lonza Bioscience (Basel, Switzerland) and characterized as before described [48, 49]. Cadaveric human islets used as control were obtained from Tebu-bio (Magenta, Italy).

### In Vitro Differentiation of HLSC into Islet-like Structures (HLSC-ILS)

HLSC were cultured at a density of 1.3E + 4/cm^2^ in a medium consisting in RPMI 1640 supplemented with 10% Fetal Bovine Serum (FBS), glucose (11.6 mM), L-glutamine (2 mM) and penicillin 100 UI/ml/streptomycin 100 μg/ml (RFG medium). To favor a charge-based cell-to-cell interaction, protamine chloride (Sigma, St. Louis, MO) was used at different concentrations: 0.1, 1 and 10 μg/ml (RFGP medium). Cells were cultured in T75 flasks (Corning, New York, NY) for 4 days with the RFG or RFGP medium, in a humidified 5% CO2 incubator at 37 °C. On day 5, the medium was completely replaced with RFG medium. Medium was subsequently changed every other day for 7, 14 and 21 days. HLSC-ILS were then counted, as previously described by Molano et al., for human islets [50].

In order to assess whether cell-density could have a role in ameliorating the efficiency of the differentiation protocol, we performed experiments by seeding HLSC in a RFG medium supplemented with protamine chloride at the most efficient concentration (10 μg/ml; RFGP) with the following different cell densities: a) 1.3E + 4/cm^2^, b) 2.6E + 4/cm^2^ and c) 3.9E + 4/cm^2^. Cells were cultured for a period of 4 days, without changing the RFGP medium, in a humidified 5% CO2 incubator at 37 °C. On day 5, the medium was replaced with RFG medium; such medium was subsequently changed every other day for a period of 14 days. HLSC-ILS were then counted [50] and viability assessed by fluorescein diacetate (FDA; Thermo Fisher Scientific, Milan, Italy) and propidium iodide (PI; Sigma).

In order to test whether glucose could have a role on structure formation, HLSC were cultured at a density of 1.3E + 4/cm^2^ for a period of 14 days in a humidified 5% CO2 incubator at 37 °C in a medium consisting in RFGP medium. On day 5, the medium was replaced with the RFG medium in absence or presence of glucose 5.6 mM, 11.6 mM or 28 mM that was subsequently changed every other day. At the end of the experiments HLSC-ILS were counted. Once the efficient concentration of protamine and cell density was established, we assessed the kinetic of HLSC-ILS formation. Specifically, HLSC with a cell density of 1.3E + 4/cm^2^ were cultured in the RFGP medium for 2 and 4 days and the total number of HLSC-ILS per condition was assessed. On day 4, the medium was replaced with the RFG medium that was subsequently changed every other day for a period of 7 and 14 days and where the number of HLSC-ILS were counted.

### Size Frequency Distribution

Micrographs were obtained using a Cell Observer SD-ApoTome laser scanning systems (Carl Zeiss International, Jena, Germany) using a 10X objective. Almost 500 HLSC-ILS were randomly selected from each of six different experiments and the major diameter was measured for each structure as described by Ricordi et al. [51]. Thereafter, the size frequency distribution of the diameters was calculated using the AxioVision software Rel3.4 (Zeiss, Germany).

### Scanning Electron Microscopy

HLSC-ILS were fixed in 2.5% glutaraldehyde in 0.1 M cacodylate (pH 7.4) as previously described [48, 52]. Samples were postfixed in 2.5% glutaraldehyde, and further dehydrated in alcohol, dried, and coated with gold by sputter coating. The specimens were observed in a scanning JeolT300 electron microscope (Jeol, Tokyo, Japan). Images were acquired at a working distance of 15–25 mm and at an accelerating voltage of 20–25 kV via secondary electron [48, 52].

### Transmission Electron Microscopy

Transmission electron microscopy of tissue specimens was carried out in Karnovsky’s-fixative, followed by treatment with osmium tetraoxide and embedding in epoxy resin according to the standard procedures [53]. Ultra-thin sections were stained with uranyl acetate and lead citrate and were analyzed with a Jeol JEM 1010 electron microscope (Jeol).

### Immunocytochemistry

We assessed by immunofluorescence the expression of pancreatic in HLSC-ILS cultured for 7, 14 and 21 days in the RFGP and/or RFG medium. At the end of the experiments HLSC-ILS were fixed in paraformaldehyde 4% (PAF, overnight), then in 30% of sucrose (overnight), included in Tissue-Tek-II (Bio-optica, Milan, Italy) and frozen at −80 °C. Cryostat sections (3–5 μm) were serially performed and fixed in acetone. In some experiments, some HLSC-ILS were disaggregated in single cells by using Trypsin 1X (Sigma) and fixed in PAF 4%. Briefly, tissue sections or cells after washing with PBS 1X were incubated for 30 min at room temperature and permeabilized with PBS 1X and Triton X-100 (0.25%), washed twice with PBS 1X for 5 min, incubated for 20 min with a blocking solution containing PBS 1X, Tween (0.1%), and 0.1% bovine serum albumin (wt/vol) and then incubated overnight with specific antibodies or irrelevant isotype controls. The following primary antibodies anti-human proteins were used: PDX1 (1:500), Ngn3 (1:50), MafB (2 μg/ml), Nkx6.1 (1:50), Nkx6.3 (2 μg/ml), chromogranin A (CgA) (2 μg/ml), C-peptide (2 μg/ml), glucagon (1:50), somatostatin (2 μg/ml), pancreatic polypeptide (3 μg/ml) and ghrelin (3 μg/ml) (1:200) (Abcam, Cambridge, MA); insulin (1:200, Dako, Copenhagen, Denmark); Glut-2 (1:200, Santa Cruz, Santa Cruz, TX). An appropriate isotopic irrelevant antibody (Abcam) was used as control. Following washing with PBS-Tween solution, sections were incubated with an appropriate secondary antibody 1:1000 (Alexa Fluor 488 Donkey anti-goat IgG for PDX1 and PP; Goat anti-Mouse IgG for CgA, C-peptide, ghrelin; Goat anti-Rabbit IgG for MafB, Nkx6.1, Nkx6.3, Glut-2, glucagon, somatostatin, Goat anti-Guinea pig for insulin; Texas Red Goat anti-Mouse IgG for Ngn3 (Invitrogen, Carlsbad, CA) at room temperature for one hour, washed with PBS-tween solution and incubated 10 min with Hoechst (Dako). After a washing with PBS, slides were mounted with Fluoromount (Sigma). Specificity of the primary antibodies recognizing human markers was verified by omitting the primary antibodies or by substitution with non-immune isotopic control. Confocal microscopy analysis was performed using a Confocal Microscope (Zeiss LSM 5 Pascal Model, Carl Zeiss International).

### FACS Analysis of Stem, Hepatic and Pancreatic Markers

In order to assess the expression of the cytoplasmic markers, the immunostainings were performed after fixation and permeabilization of HLSC and HLSC-ILS dissociated cells with Trypsin-EDTA (0.1%) and analyzed by FACS. Cytofluorimetric analysis was performed as previously described (24-Cabrera). We used the following antibodies, that were phycoerythrin (PE) or fluorescein isothiocyanate (FITC) or allophycocyanin (APC)-conjugated: anti-CD105, -CD31, -CD29, -CD44 (Dako Denmark A/S, Copenhagen, Denmark); -CD90, -CD73 (BD Biosciences Pharmingen, San Jose); and unconjugated antibodies (1:20) anti-albumin, −synaptophysin (Dako), -PDX1, c-peptide, −glucagon, -Ngn3 (Abcam); Alexa fluor 488-conjugated goat antibodies against mouse/rabbit/pig IgG (Dako) were used as secondary antibodies (1:1000) when needed. Briefly, the cells were resolved in PBS containing 0.1% bovine serum albumin or PBS containing 0.1% Triton to permeabilize cells and 0.1% bovine serum albumin. All the incubations were performed at 4 °C for 1 h. After incubation cells were washed twice., Ten thousands cells for each sample were examined by FACScan cytometer (BD Biosciences Pharmingen, San Diego, CA). Compensation controls were included in all the analyses performed and gating was constructed based on negative controls. For gated populations percentages and numbers were generated from each experiment using Cell Quest software (BD Biosciences Pharmingen). Results were calculated as percentage of positive cells and as geometric mean of fluorescence.

### Western Blot Analysis

HLSC and HLSC-ILS were lysed at 4 °C for 1 h in RIPA buffer added with phosphatase and protease inhibitors (Sigma) and centrifuged at 15,000 g. After addition of lysis buffer, HLSC structures were subjected to 2 cycles of 30 s of sonication with a pause of 10 s on ice. Sonication was necessary to obtain disintegration of the capsule enveloping the structures. Aliquots containing 30 μg of proteins were quantified by Bradford method, loaded in a 4–15% gradient Tris HCl gel electrophoresis under reducing conditions and electro-blotted onto nitrocellulose membranes. The membranes were blocked with non-fat milk and overnight incubated with primary antibodies: anti-C peptide (Abcam); anti-Glucagon (Abcam) and anti-Actin (Santa Cruz Biotechnology) at the appropriate concentration. After extensive PBS-T washings, the membranes were incubated with the specific secondary antibodies (Thermo Fisher Scientific) for 1 h at room temperature, washed with PBS-T, developed with ECL detection reagents, and subjected to Chemidoc (Bio-Rad, Hercules, CA).

### RT-PCR Analysis

RNA was extracted by Trizol (Life Technologies, Thermo Fisher Scientific) and 200 ng of total RNA were retrotranscribed using High Capacity cDNA Reverse Transcription Kit (Applied Biosystems, Thermo Fisher Scientific). 16 ng of cDNA and 300 nM of each primer were added to the RT-PCR mix containing 1X Power SYBR GREEN PCR Master Mix (Applied Biosystems) and were run on 96-well StepOne Real Time System (Applied Biosystems). No template controls (no cDNA) were cycled in parallel with each run. GAPDH and ACTIN were used as housekeeping gene.

### RNA Extraction

HLSC and Islets were harvested from flasks and centrifuged at 900 rpm for 10 min. Supernatant was discarded and pellet was washed with PBS and centrifuged at 900 rpm for 10 min. Supernatant was discarded and pellet was resuspended in 200 μl of RNase free water (Ambion) plus 5 μl of RNAse Inhibitor (Ambion, Thermo Fisher Scientific). Cell suspension was cooled on ice and then islets, but not HLSC, were sonicated at 30% potency for 30 s two times. Then 750 μl of TriZol LS (Life Technologies) were added to each sample and RNA was extracted according to manufacturer’s instructions. RNA concentration and quality were measured by Nanodrop ND-1000 (Thermo Fisher Scientific).

### Microarray Gene Expression Analysis

500 ng of total RNA were retro-transcribed to cDNA with SuperScript IV VILO Master Mix with ezDNase enzyme (Invitrogen, Thermo Fisher Scientific) following kit’s instructions and by using Veriti Thermal Cycler (Applied Biosystems). The expression profile of a panel of 45 target genes selected among HLSC markers, β cell markers, and genes involved in glucose metabolism was tested [54]. For each selected target gene plus 3 endogenous controls, the specific TaqMan® Gene Expression Assay (Applied Biosystems) was spotted on a custom TaqMan® Array microfluidic cards (Applied Biosystems) (supplementary Table I). The samples were mixed with TaqMan® Fast Advanced Master Mix (Applied Biosystems), TaqMan® Array microfluidic cards were prepared according to manufacturer’s instruction and run with the QuantStudio 12 K Flex Real-Time PCR System (Applied Biosystems). Each sample was run in triplicate and 3 samples for each experiment were tested. Retrotranscription negative controls (no RT enzyme) were run for 6 TaqMan® Gene Expression Assay selected among the 48 spotted on the TaqMan® Array microfluidic cards. The run was performed in a 96 well plate by QuantStudio 12 K Flex Real-Time PCR System (Applied Biosystems). No template control (no cDNA) was run once on TaqMan® Array microfluidic cards. Both negative controls showed no amplification at Ct < 37. ExpressionSuite Software 1.0.3 (Thermo Fisher Scientific) was used to calculate RQ (2^^-∆∆Ct^) values. B2M, GUSB, and PPIA were set as endogenous controls for multiple endogenous control analysis. Data were normalized to HLSC expression level.

### In Vitro Static Glucose-Stimulated Insulin Release Assay

After a culture period of 14 days in the RFGP and/or RFG medium, the HLSC-ILS were cultured overnight in a medium consisting in DMEM (5.6 mM) supplemented with FBS (10%), L-glutamine (2 mM) and penicillin 100 UI/ml/streptomycin 100 μg/ml. Thereafter, a total of 400 IEQ were placed in 12 well-culture plates and cultured in Krebs buffer with a low concentration of glucose (2.8 mM) for 1 h; the medium was subsequently collected and replaced with a Krebs buffer supplemented with glucose (28 mM). Cells were then placed in a humidified 5% CO2 incubator at 37 °C for 1 h. The supernatant was then collected and replaced with Krebs buffer supplemented with glucose 2.8 mM and KCl (50 mM). Cells were then placed in a humidified 5% CO2 incubator at 37 °C for 1 h and the supernatant was then collected. All the supernatants were stored at −20 °C for subsequent analysis using an ultrasensitive human C-peptide enzyme-linked immunosorbent assay kit (Alpco Diagnosis, Windham, NH), according to the manufacturer instructions. Total protein content within the structures was assessed by Bradford method in order to adjust human C-peptide concentration.

### Dynamic Glucose-Stimulated Insulin Release Assay

HLSC-ILS 14 days post-differentiation (400 IEQ) were seeded in a cell culture Microchip (Micronit Microfluidics, Netherlands) and incubated at 37 °C for 3 h in DMEM 10% FCS (5.6 mM of glucose) prior to microfluidic stimulation. Then, islets were exposed to a flow rate of 30 μl/min generated by a flow controller platform (MFCS-EZ) using MAESFLO 3.3.4 Software (Fluigent, France). The glucose/KCl pulse stimulation study was based on Oberholzer’s group protocol (55-Mohammed) for testing physiological function of islets with some modifications: 0) media wash for 10 min, 1) low glucose (2.8 mM) for 10 min, 2) high glucose (28 mM) for 30 min, 3) low glucose (2.8 mM) for 10 min, 4) KCl (50 mM) for 15 min, and finally 5) low glucose (2.8 mM) for 10 min. Each step was preceded by a transition state ranging from 10 to 20 min to change between different concentrations of glucose and KCl solution. At the end of each experiment, perfusate samples were collected in individual tubes, centrifuged at 900 rpm for 5 min to eliminate cell debris, the supernatants were transferred into new tubes and stored at −20 °C prior to analysis. HLSC-ILS were detached from the culture chip, total protein was extracted (see Western blot analysis methods) and samples were stored at −20 °C prior to analysis. The hC-peptide was assessed by ELISA (ALPCO Diagnosis, Windham, NH) according to the manufacturer’s instructions, while total protein was quantified by BCA colorimetric detection assay (BCA protein assay kit, Pierce). The hC-peptide secretion was normalized by protein content.

### Experimental Animals

Animal studies were approved by the Ethics Committee of Istituto Superiore di Sanità (741/2015-PR) and performed according to the National Institute of Health Guide for the Care and Use of Laboratory Animals. 8–9 weeks old male SCID mice obtained from Charles Rivers (Calco, Italy) were maintained in sterile conditions and supplied with food and water ad libitum.

### Induction of Diabetes

Diabetes (glycemia >250 mg/dl) was induced in SCID mice weighing ~22 g by intraperitoneal injections of streptozotocin (STZ; Sigma)-citrate buffer (55 mg/kg/day) for 5 consecutive days (53-Breyer). Sham mice injected with sodium citrate buffer were used as controls. Blood glucose was measured between 2:00 p.m. and 4:00 p.m. via tail vein sampling on alert, fasted animals, using the diagnostic glucose oxidase enzymatic test (Glucometer Accu-Chek, Roche, Mannheim, Germany). Groups of diabetic SCID mice with similar blood glucose levels and nondiabetic control mice were studied in parallel and divided in the following subgroups: a) nondiabetic control SCID mice; b) STZ-induced diabetic SCID mice (DM); c) DM mice that received HLSC-ILS under the right renal capsule (DM + HLSC-ILS).

### HLSC-ILS Implantation under the Kidney Capsule

HLSC-ILS cultured for a period of 14 days were counted and scored for size, according to the algorithm previously described for the calculation of the 150 μm diameter islet equivalent (IEQ) number [51]. Diabetic SCID mice were anesthetized by intramuscular injection of zolazepam (Zoletil; VIRBAC, Milan, Italy) (0.2 ml/Kg) and xilazine (Rompun; Bayer. Milan, Italy) (16 mg/Kg). Subsequently, under aseptic conditions, the right kidney was carefully externalized through a dorsal lumbotomy incision and the HLSC-ILS (800 IEQ) delivered under the renal capsule with a 24 GA catheter. The kidney was then repositioned in the retroperitoneal space and the skin incision closed using a 6–0 silk suture. Following islets-like structures implants, blood glucose obtained via tail vein sampling between 2:00 p.m. and 4:00 p.m. on alert 4 h–fasted animals was measured weekly (Glucometer Accu-Chek, Roche) with a maximum follow-up period of 7 weeks.

### Intraperitoneal Glucose Tolerance Test (IPGTT)

Intraperitoneal glucose tolerance tests (IPGTT) was performed in some non-diabetic (non-DM), diabetic (DM) mice, and DM mice implanted with HLSC-ILS. In the last group, IPGTT was performed before (pre-explant) and after the implant removal (post-explant). Blood glucose was measured between 2:00 p.m. and 4:00 p.m. on alert 4 h–fasted animals via tail vein sampling and analyzed by glucometer (Accu-Chek, Roche, Mannheim, Germany). For IPGTT, blood glucose was measured immediately before (T0) and at 15, 30, 60 and 120 min after an IP glucose (2 g/kg in 0.9% NaCl) injection.

### Surgical Removal of HLSC-ILS Implants (Explant)

At the end of the experiments, the implants were removed from normoglycemic implanted animals. Briefly, under aseptic conditions, we performed an abdominal lobotomy incision, the right kidney was carefully exposed and the portion of renal capsule containing the structures removed to explant the HLSC-ILS. The kidney was then repositioned in the retroperitoneal space and the muscle and skin incisions closed using a 6–0 silk suture. One week after the explant, glycemia was assessed in blood samples obtained from the tail vein between 2:00 p.m. and 4:00 p.m. on alert 4 h–fasted animals (Glucometer Accu-Chek, Roche).

### In Vivo Assessment of Human C-Peptide Serum Concentration

Mice were sacrificed at week 8 post-implant; blood samples were collected and serum separated. The human C-peptide levels were assessed by using an ultrasensitive human C-peptide enzyme-linked immunosorbent assay kit (Alpco Diagnosis, Windham, NH) according to the manufacturer instructions.

### Histology and Immunohistochemical Analysis of HLSC-ILS Explants

Animals were euthanized under anesthesia by exsanguination via cardiac puncture, the right kidney was removed, fixed in 10% formalin (vol/vol) and processed for histology. Hematoxylin and eosin (H&E) staining was performed on 4 μm kidney paraffin sections.

The expression of human C-peptide and glucagon in the implants were assessed by immunofluorescence using specific antibodies. Sections were dewaxed and hydrated, boiled in citrate buffer (1X) at 100 °C for 20 min and then incubated with PBS-0.1% Tween-0.1% bovine serum albumin (wt/vol) for 30 min at room temperature to block the unspecific reactivity. Subsequently, sections were incubated overnight with the specific antibodies: anti-human C-peptide and anti-human glucagon (1:200; Abcam). In the negative control the primary antibodies were omitted or substituted with non-immune human IgG. Then the sections were incubated with the corresponding secondary antibodies (1:500 Goat anti Rabbit Alexa Fluor 488 and Goat anti mouse Texas Red (Invitrogen), subsequently with Hoechst 1:500 (Dako) for 10 min and then mounted with Fluoromount (Sigma). Microscopy analysis was done using a Cell Observer SD-ApoTome laser scanning systems with 20X objective (Carl Zeiss). Analysis was performed in a blinded fashion with AxioVision 4.8 software (Carl Zeiss).

### Insulin RNA Fluorescence In Situ Hybridization (FISH)

Formalin fixed explants were de-paraffined and 3 μM slides were processed. FISH was made according to the manufacturer standard protocol (Biosearch Technologies). Briefly, probes mixture at 4–5 nM (100 nM for tissue sections, equimolar mixture of all probes) was dissolved in hybridization buffer (10% dextran sulfate, 0.02% BSA, 2 × SSC, 10% formamide, 2 mM vanadyl-ribonucleoside complex) and added to tissue sections and incubated overnight at 37 °C. Cell nuclei were stained with 0.05% Hoechst.

### Statistical Analysis

The number of experiments is properly reported in figure legends. All data were analyzed with GraphPad Prisma software and presented as mean ± standard deviation (SD). Student’s t test was used for the comparison between two groups. ANOVA was used when more than two groups were studied, and the Newman–Keuls, or Dunnet, or Kruskal-Wallis multi-comparison tests were used when appropriate. *p* values <0.05 were considered statistically significant.

## Results

### Protamine Induces HLSC to Form 3D Spheroid Structures

**Fig. 1:**
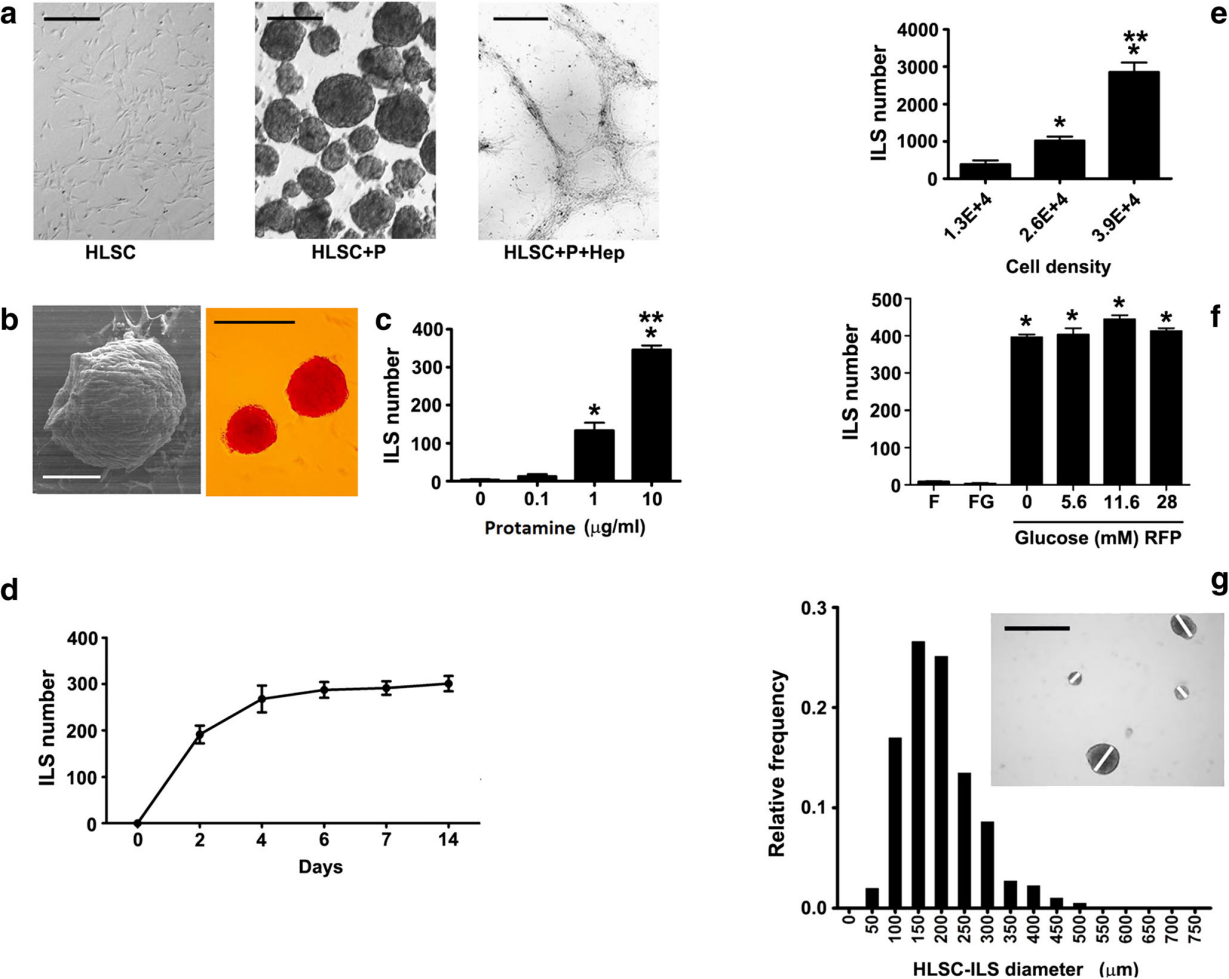
In vitro differentiation of HLSC into islet-like structures (HLSC-ILS). **a** Representative micrographs of 10 experiments showing 1.3E + 4/cm^2^ non-stimulated cells (HLSC) and cells stimulated with protamine 10 μg/ml (HLSC+P) or with 10 IU/ml heparin –protamine (10 μg/ml) (HLSC+*p* + Hep). Scale bars = 200 μm. **b** Representative micrographs of scanning electron microscopy (left; scale bar = 100 μm) and of dithizone staining (right; Scale bars = 200 μm) of HLSC-ILS showing their characteristic phenotype. **c** Dependence of HLSC-ILS generation on protamine concentrations (*n* = 6). **d** Growth curve of HLSC-ILS (n = 6). **e** Dependence of HLSC-ILS generation on cell density (cells/cm^2^) (n = 6). **f** Contribution of glucose and FBS to the generation of HLSC-ILS. RPMI basal medium (R), fetal bovine serum (F), glucose (G) and protamine (P) (n = 6). **g** Size frequency distribution of the mean profile diameter of HLSC-ILS expressed as relative frequency (Scale bars = 300 μm). The insert shows an example of the diameter measurement**.** Data are expressed as media ± SE of three independent experiments. ANOVA with Dunnet’s multi-comparison test was performed; *p < 0.01 versus 0 (C), 1.3E + 4 (E) or versus F and FG (F)

As shown in Fig. 1a, HLSC cultured in a RGFP/RFG medium enriched with protamine chloride (HLSC+P) resulted in a fast formation of numerous HLSC-ILS structures. Such effect was blunted after the neutralization of protamine with heparin (HLSC+*p* + Hep). Scanning electron microscopy showed 3D spheroidal structures that were positive for dithizone staining (Fig. 1b). The addition of protamine was followed by cell cluster formation that was dose dependent (Fig. 1c) and time-dependent (Fig. 1d), with a maximum effect after a culture period of 14 days in the presence of a protamine concentration of 10 μg/ml. Based on these results, in subsequent experiments we used a protamine concentration of 10 μg/ml. As shown in Fig. 1e, structure formation was also cell density-dependent. Indeed, HLSC-ILS number was significantly higher in the presence of a basal cell density of 3.9E + 4/cm^2^ after a culture period of 14 days. On the other hand, neither FBS nor glucose concentration did appear to impact on cell cluster formation, as HLSC-ILS number was similar in the absence or in the presence of different glucose concentrations (5.6 mM, 11.6 mM or 28 mM) or FBS 10% (Fig. 1f).

The morphometric analysis demonstrated that the diameter of HLSC-ILS after a culture period of 14 days (Fig. 1g) was comprised between 100 and 250 μm, with an average islet volume of 194 ± 79.6 μm (mean ± SD; median 181.9 μm) as comparable to that reported for human pancreatic islets [51]. The size of HLSC-ILS was not significantly different after a culture period of 7 (144.1 ± 61.0) and 21 (224.0 ± 107.0) days.

**Fig. 2:**
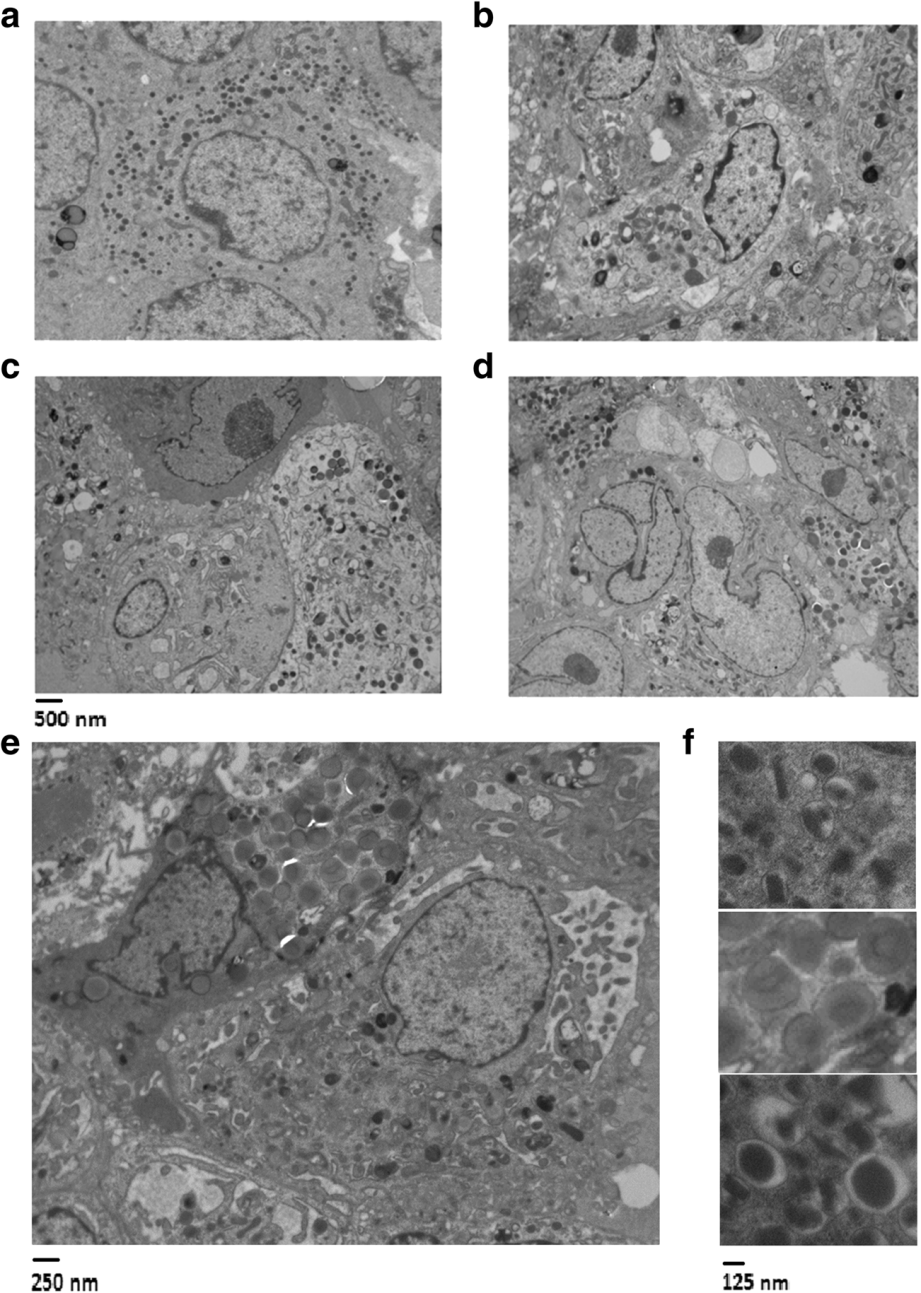
Representative transmission electron microscopy of cadaveric human islets (**a**) and HLSC-ILS structures formed after 4 (**b**), 7 (**c**) and 14 (**d**) day stimulation with 10 μg/ml protamine. Heterogeneity of granules in HLSC-ILS at 14 days at higher magnification (**e** and **f**)

By transmission electron microscopy, endocrine granules typical of human islets (Fig. 2a) were observed in the 3D spheroidal structures at day 4, 7 and 14 of differentiation (Fig. 2b–d). As seen in Fig. 2e, f, three main types of electron-dense granules were observed: granules containing crystalloids, granules with a diffuse gray pale core, and granules with a dense core.

### HLSC-ILS Cell Composition and Expression of Typical Endocrine Pancreatic Factors

**Fig. 3:**
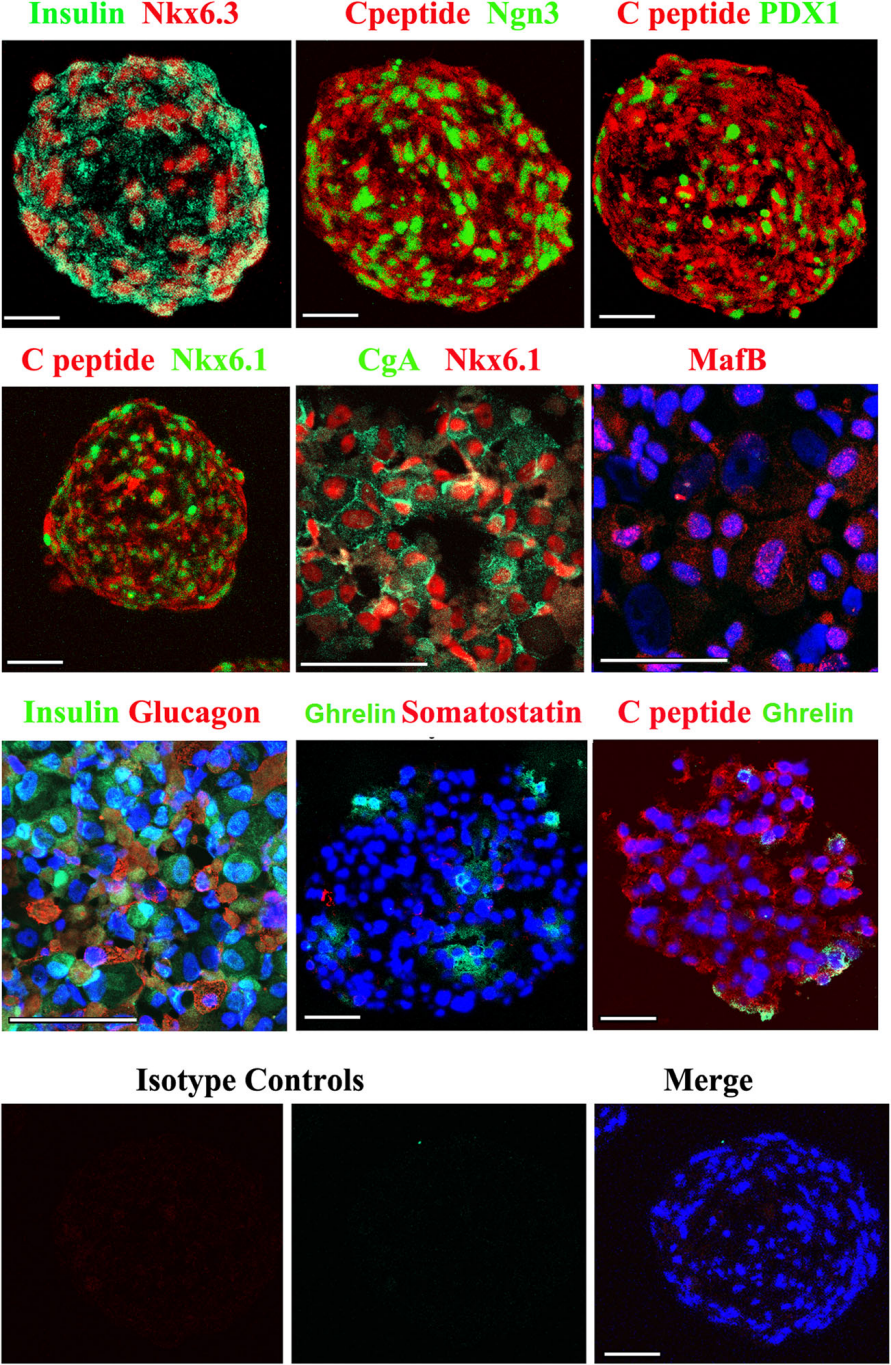
Representative immunofluorescence for β-cell transcription factors and pancreatic hormones in cryostat sections of HLSC-ILS. The appropriate irrelevant isotype control antibodies used as controls were all negative. Original magnification ×400 (scale bars = 50 μm). Images are representative of 10 experiments performed with similar results

By immunofluorescence HLSC-ILS expressed the β-cell transcription factors Nkx6.1, Nkx6.3, MafB and the endocrine specific markers chromogranin A (CgA) and Ngn3 at 14 days of differentiation (Fig. 3). Furthermore, at the same time-point HLSC-ILS expressed the exocrine pancreatic marker PDX1 and the human islet specific hormones, including insulin/C-peptide, glucagon, somatostatin and ghrelin (Fig. 3). All isotype controls were negative. Similar but less intense positivity was detectable at 4 and 7 days of differentiation (data not shown).

**Fig. 4:**
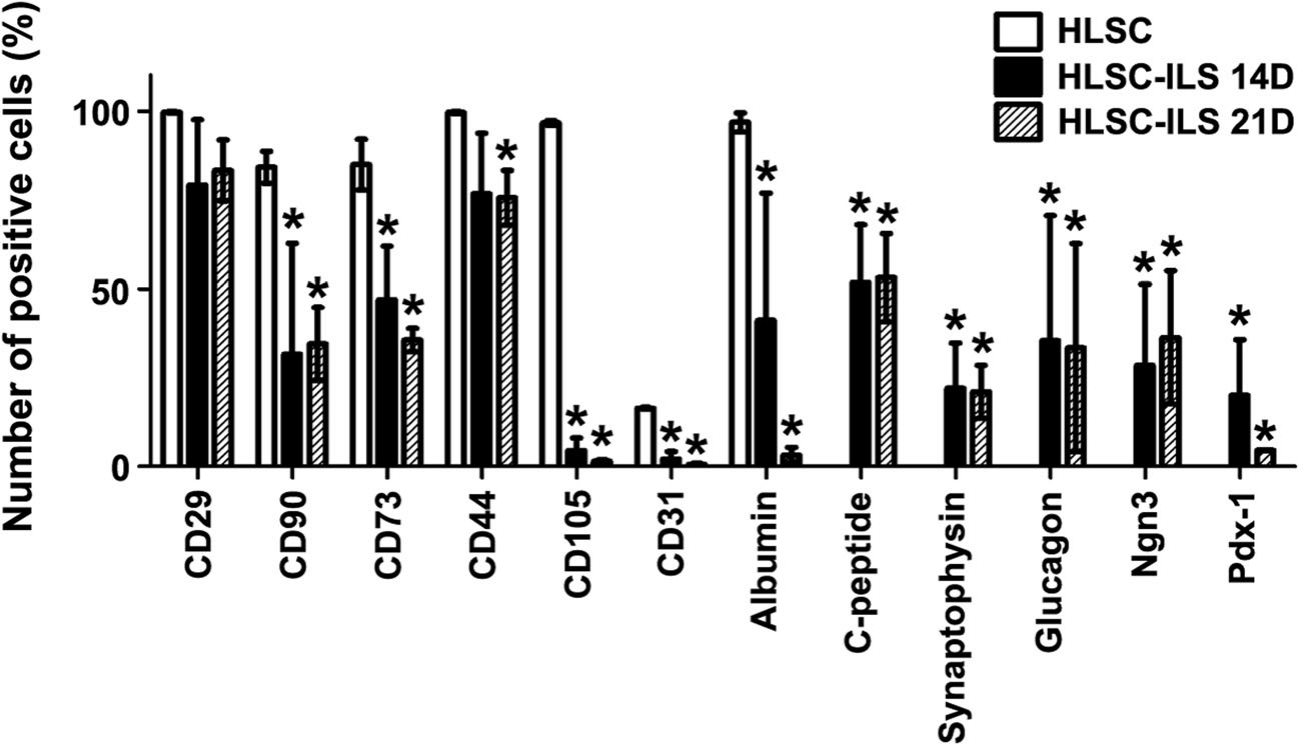
Flow cytometry analysis of stem cells markers (of CD73, CD90, CD105, CD31), albumin and islets specific markers (PDX1, Ngn3, synaptophysin, C-peptide and glucagon) in HLSC and HLSC-ILS. Three independent experiments were performed with similar results and are expressed as media ± SE, ANOVA with Newman–Keuls multi-comparison test was performed; *p < 0.05 versus HLSC

FACS analysis on cell populations isolated after dissociation of HLSC-ILS, confirmed the expression of islet specific markers (i.e. PDX1, Ngn3, synaptophysin, C-peptide and glucagon) and showed a down-regulation of HLSC markers (i.e. CD90. CD73, CD105, CD31) and albumin (Fig. 4).

**Fig. 5:**
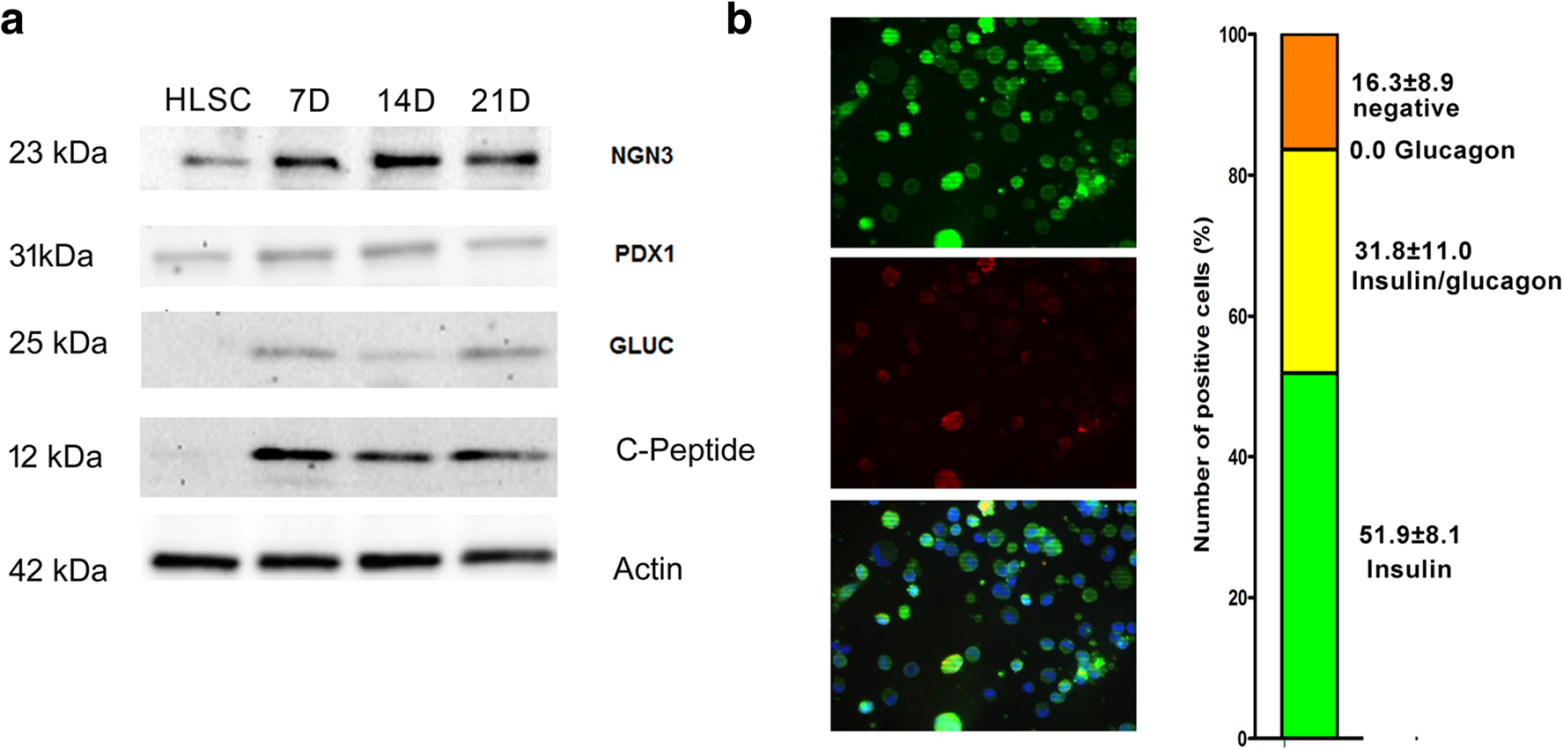
Phenotypic characterization of non-differentiated HLSC (HLSC) and differentiated HLCS-ILS at different times of culture (7, 14 and 21 days). **a** The expression of human Ngn3, PDX1, C-peptide and glucagon were analyzed by Western Blot. **b** Representative micrographs of immunofluorescence and percentage of different cell subpopulations expressing insulin alone, insulin and glucagon and negative for both hormones

The expression of Ngn3, PDX1, C-peptide, glucagon and somatostatin proteins was confirmed also by Western Blot analysis of HLSC-ILS cultured for 7, 14 and 21 days (Fig. 5a). Non-differentiated HLSC expressed low levels of Ngn3 and PDX1. In contrast, C-peptide, glucagon and somatostatin were observed only in differentiated HLSC-ILS from day 7 to day 21.

To characterize cells co-expressing hormones, HLSC-ILS were dissociated into single cells and stained by immunofluorescence. Insulin-expressing cells were 51.9% ± 8.1, insulin/glucagon co-expressing cells were 31.8% ± 11.0, and negative cells for both hormones were 16.3 ± 8.9% (Fig. 5c). No cells expressing only glucagon were detected. The presence of cells positive for somatostatin, ghrelin and PP was less than 1%.

**Fig. 6:**
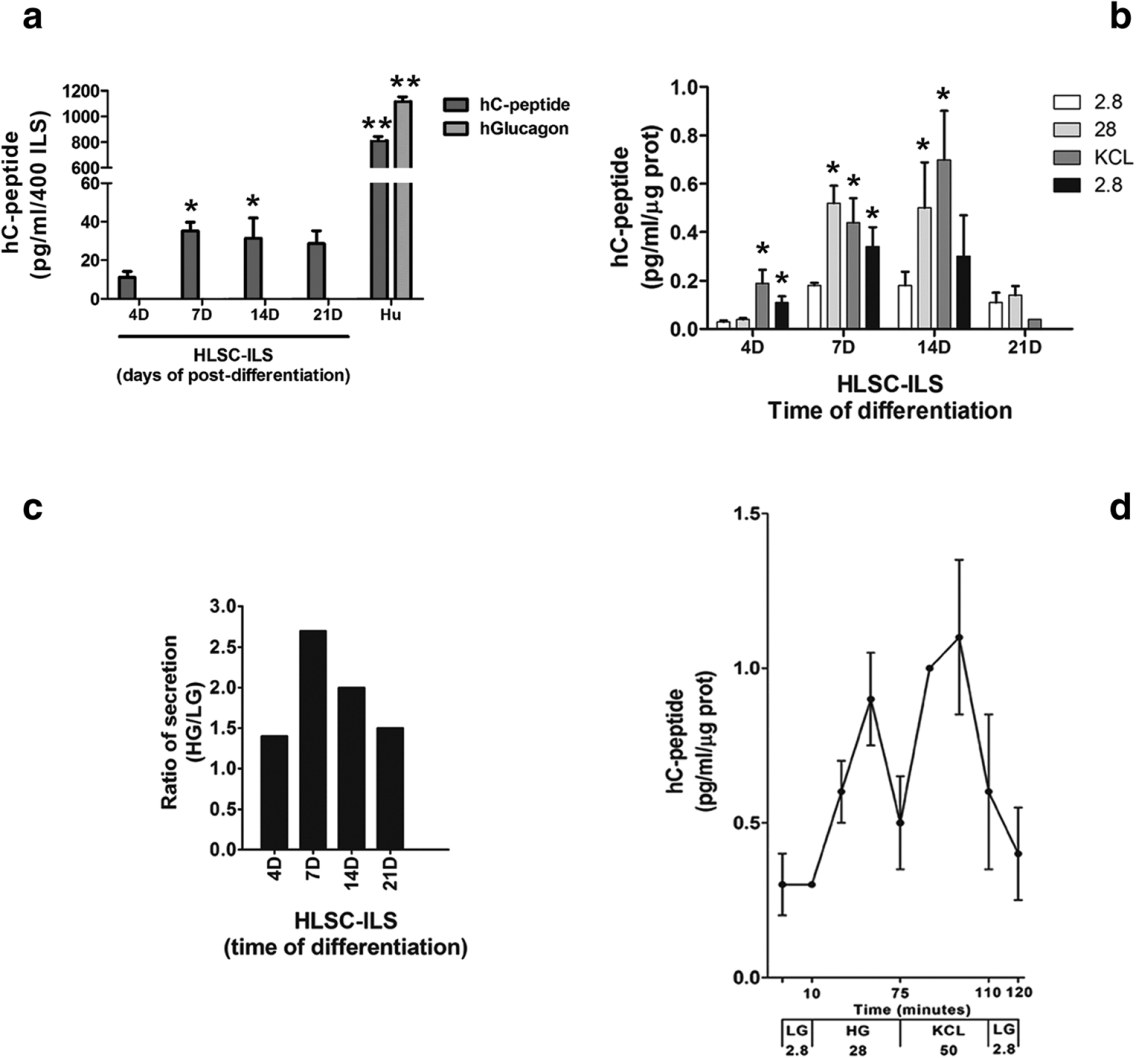
**In vitro hC-peptide secretion analysis in static and dynamic conditions. a** In vitro human hC-peptide and glucagon basal secretion by HLSC-ILS (4, 7, 14 and 21 days) was evaluated in static condition and compared with human islets. A total of 400 HLSC-ILS or cadaveric human islets were stimulated with 2.8 mM of glucose. Dunnet’s multi-comparison test was performed; data are presented as mean ± SD of 3 independent experiments. *p < 0.001 versus HLSC-ILS 4D and **p < 0.001 versus HLSC-ILS. **b** Static in vitro hC-peptide secretion by HLSC-ILS (4, 7, 14 and 21 days) after stimulation with two different concentrations of glucose (2.8 and 28 mM) and 50 mM of KCl. (**c**) HG/LG ratio secretion of hC-peptide of HLSC-ILS (4, 7, 14 and 21 days). Dunnet’s multi-comparison test was performed; data are presented as mean ± SD of 3 independent experiments *p < 0.001 versus 2.8 mM. **C)** HG/LG ratio of secretion by HLSC-ILS (4, 7, 14 and 21 days) and (**d**) Dynamic secretion of hC-Peptide by HLSC-ILS at 14 days of differentiation stimulated by 2.8 mM of glucose (LG), 28 mM of glucose (HG) and high potassium (KCl). The concentration of hC-peptide was assessed by ELISA and normalized to total protein. Data are expressed as mean ± SD of 4 independent experiments

Compared with not-differentiated HLSC cells, a basal secretion of human C-peptide was detectable in HLSC-ILS at 4 days post-differentiation, increased at day 7 and maintained until day 21(Fig. 6a). However, the level of C-peptide basal secretion was markedly inferior to that of human islets (Fig. 6a). The secretion of glucagon was not detected (Fig. 6a).

### HLSC-ILS Secrete Human C-Peptide in Response to Glucose in both Static and Dynamic Conditions

The glucose-induced secretion was evaluated in the HLSC-ILS at day 4, 7, 14 and 21 of differentiation by static incubation with 2.8 mM low glucose (LG) and 28 mM high glucose (HG) and 50 mM KCl. HLSC-ILS showed a basal secretion of hC-peptide but did not respond to both LG and HG at day 4 of differentiation. A slight increase in secretion was observed in response to KCl (Fig. 6b). In contrast, the basal levels of hC-peptide were increased at 7 and 14 days. Secretion of hC-peptide increased after HG stimulation (Fig. 6b). KCl induced a further secretion only in the HLSC-ILS at 14 days of differentiation (Fig. 6b). The HG/LG secretion ratio (Fig. 6c) demonstrated an enhanced response to glucose at 7 and 14 days (2.5 and 2.0, respectively) compared to day 4. The secretion in response to HG or KCl stimulation was reduced at day 21 (Fig. 6b–c).

The kinetic of secretion was evaluated at 14 days of differentiation, by using a dynamic microfluidic system. The kinetic of secretion showed an initial basal secretion to LG, followed by two peaks of secretion in response to HG and KCl (Fig. 6d). Each peak was reduced when we shifted to LG. The ratio of secretion was 4.5 and 5.6 in response to HG and KCl, respectively.

### HLSC-ILS Show β Cell Gene Expression Commitment

**Fig. 7:**
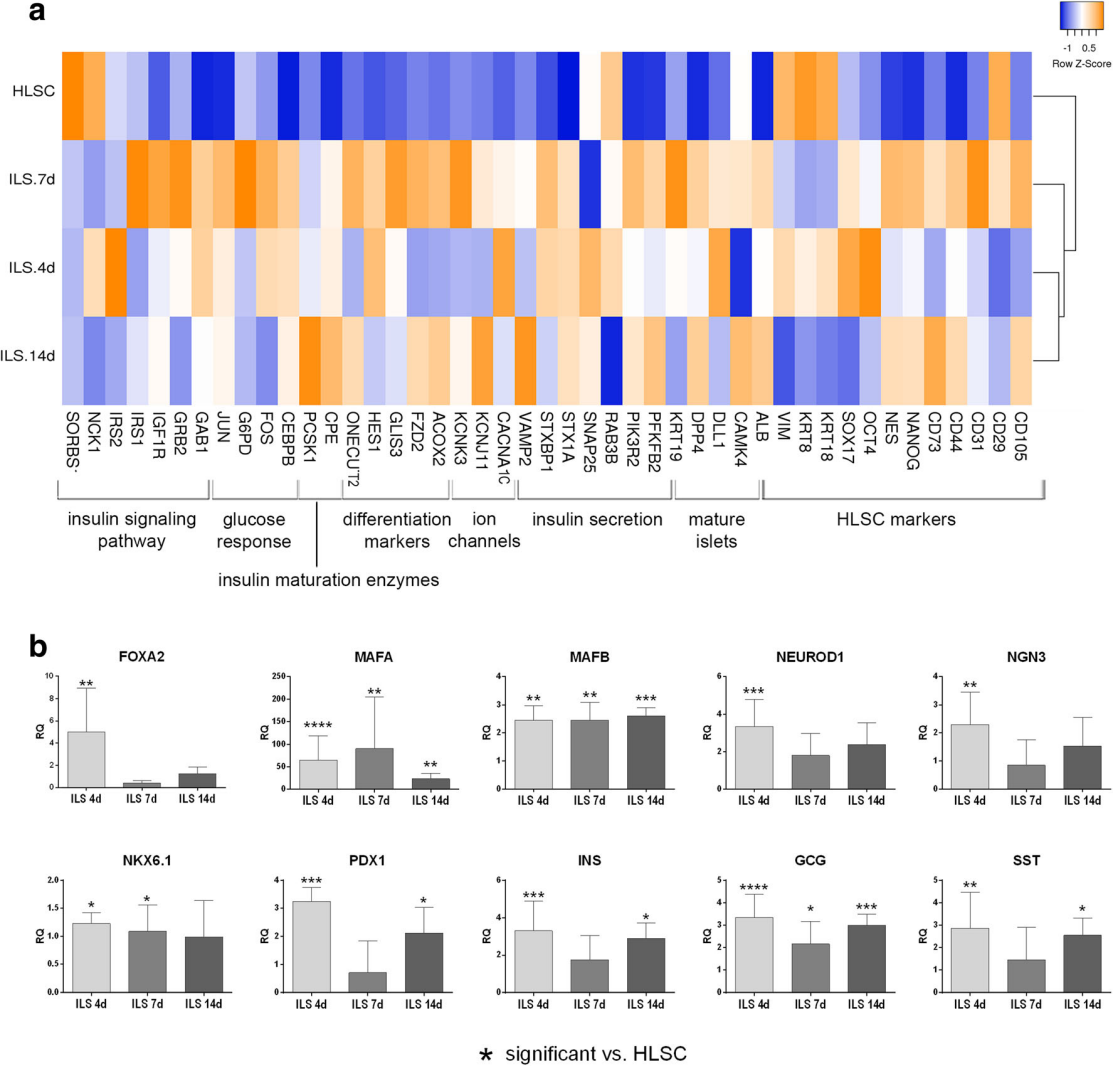
qRT-PCR array gene expression analysis of non-differentiated HLSC and differentiated HLCS-ILS at different times of culture. **a** Hierarchical clustering analysis of HLSC and HLSC-ILS at 4 (ILS 4d), 7 (ILS 7d), and 14 (ILS 14d) days of culture. The analyzed genes are listed below the heat map and grouped by gene function. The expression levels are reported as row Z score of RQ (2^^-∆∆Ct^) values (scale color: *blue* low expression, *orange* high expression) of three independent experiments run in triplicate. Average linkage clustering method and Euclidean distance measurement methods were used. The heat map was generated with heatmapper online software (http://www.heatmapper.ca/expression/). **b** Real Time PCR analysis of transcription factors involved in β cells maturation and pancreatic hormones. Values are reported as mean ± SD of RQ of three independent experiments run in triplicate. Expression is normalized for HLSC (RQ = 1, not shown). * *p* < 0.05, ** *p* < 0.01, *** *p* < 0.001, **** *p* < 0.0001

Comparative qRT-PCR microarray analysis showed that HLSC-ILS at different days of differentiation (4, 7, and 14 days) modify their gene expression profile compared to undifferentiated HLSC. The heat map in Fig. 7a shows that HLSC-ILS gene expression profiles are clustered together, with a similar expression pattern, while undifferentiated HLSC gene expression profile was clustered apart and showed a different gene expression pattern. HLSC-ILS showed a decreased expression of HLSC markers, such as cytokeratin 18 (KRT18), cytokeratin 8 (KRT8) and vimentin (VIM) and an increased expression of NANOG and nestin (NES), two stem cell markers, together with two insulin maturation enzymes and several markers involved in β cell differentiation. Moreover, we observed an increased expression of several transcripts for proteins involved in insulin granules release, such as PFKFB2, STX1A, STXBP1, VAMP2, and the calcium (CACNA1C) and potassium (KCNJ11) channels (Fig. 7a). Moreover, qRT-PCR showed an enhanced expression of several transcription factors specifically involved in β cell differentiation, such as FOXA2, MAFA, MAFB, NEUROD1, NGN3, NKX6.1, and PDX1. Furthermore, the insulin, glucagon and somatostatin mRNA expression was higher than HLSC expression at each time point (Fig. 7b).

### HLSC-ILS Rapidly Reverse Hyperglycemia in Diabetic SCID Mice

**Fig. 8:**
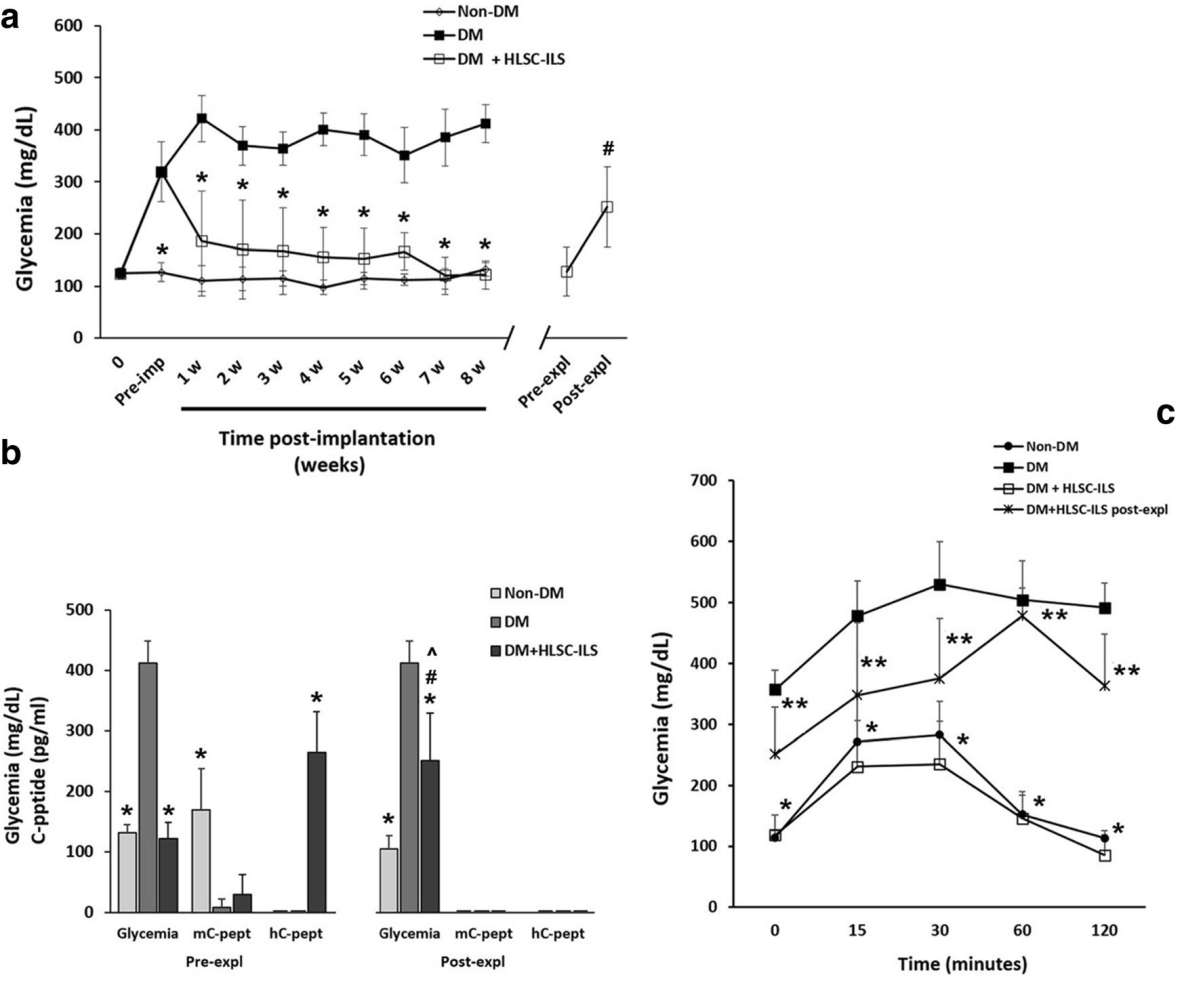
In vivo reversal of diabetes in SZT-treated SCID mice by implantation of 800 HLSC-ILS (**28,000** IEQ/kg) under the right renal capsule. **a** Blood glucose levels in sham non-diabetic (non-DM, n. 12), diabetic (DM, n. 8), DM mice implanted with HLSC-ILS (DM + HLSC-ILS, n. 12) and DM + HLSC-ILS before (Pre-expl) and after (Post-expl) explantation (n. 4). ANOVA with Newman–Keuls multi-comparison tests was performed. * *p* < 0.001 versus DM; ^**#**^*p* < 0.05 versus Pre-expl. Data are presented as mean ± SD. **b** Correlation between the serum levels of mouse and human C-peptide (mC-peptide and hC-peptide, respectively) and glycemia in non-DM, DM and DM-HLSC-ILS before (Pre-expl) and after (Post-expl) explantation. ANOVA with Newman–Keuls multi-comparison tests was performed. * *p* < 0.001 versus DM; # *p* < 0.05 glycemia DM + HLSC-ILS Pre-expl versus Post-expl; ^ *p* < 0.001 DM + HLSC-ILS Post-expl versus Non-DM Post-expl. Data are presented as mean ± SD. hC-petide was detectable only in DM + HLSC-ILS Pre-expl. mCpeptide and hC-peptide were undetectable in DM + HLSC-ILS Post-expl. **C)** IPGTT curves in non-DM (n.10), DM (n. 3) and DM + HLSC-ILS Pre-expl (n. 7) and Post-expl (n. 4). ANOVA with Newman–Keuls multi-comparison tests was performed. * ^+^*p* < 0.001 versus DM; ** *p* < 0.001 versus Pre-expl. Data are presented as mean ± SD

Intraperitoneal injection of streptozotocin was followed by induction of hyperglycemia in SCID mice (Fig. 8a). Implant of HLSC-ILS (following 14 days in vitro differentiation) under the renal capsule of diabetic SCID mice was associated with a progressive reduction of blood glucose levels which was already significant after 1 week of implantation and was sustained up to 8 weeks. When the implants were removed after 8 weeks, a significant increase of glycemia was observed, suggesting that the HLSC-ILS implants were responsible for achievement of euglycemia in recipient mice. Non-implanted diabetic mice and non-diabetic mice were used respectively as hyperglycemic and normoglycemic controls.

Changes in glycemia were directly correlated with serum levels of C-peptide (Fig. 8b). We observed that, compared to non- diabetic mice, the murine C-peptide levels were minimal or absent in both diabetic mice and diabetic mice treated with HLSC-ILS (Fig. 8b). The presence of sustained hyperglycemia in diabetic mice supports that this remnant quantity of mC-peptide is not sufficient to maintain euglycemia. Furthermore, hC-peptide was detected only in the diabetic mice implanted with HLSC-ILS and was undetectable after removing the implants.

We performed intraperitoneal glucose tolerance tests (IPGTT) to assess the in vivo glucose-stimulated insulin release (Fig. 8c). Compared with non-diabetic mice, diabetic ones showed a high basal glycemia and an altered glucose tolerance test with sustained hyperglycemia. In contrast, the HLSC-ILS-implanted diabetic mice showed normalization of basal blood glucose levels and a response to IPGTT comparable to non-diabetic control mice. Glycemia returned to basal levels after 120 min. Following explant of HLSC-ILS, mice returned to hyperglycemia and showed an altered IPGTT curve comparable to diabetic mice (Fig. 8c).

**Fig. 9:**
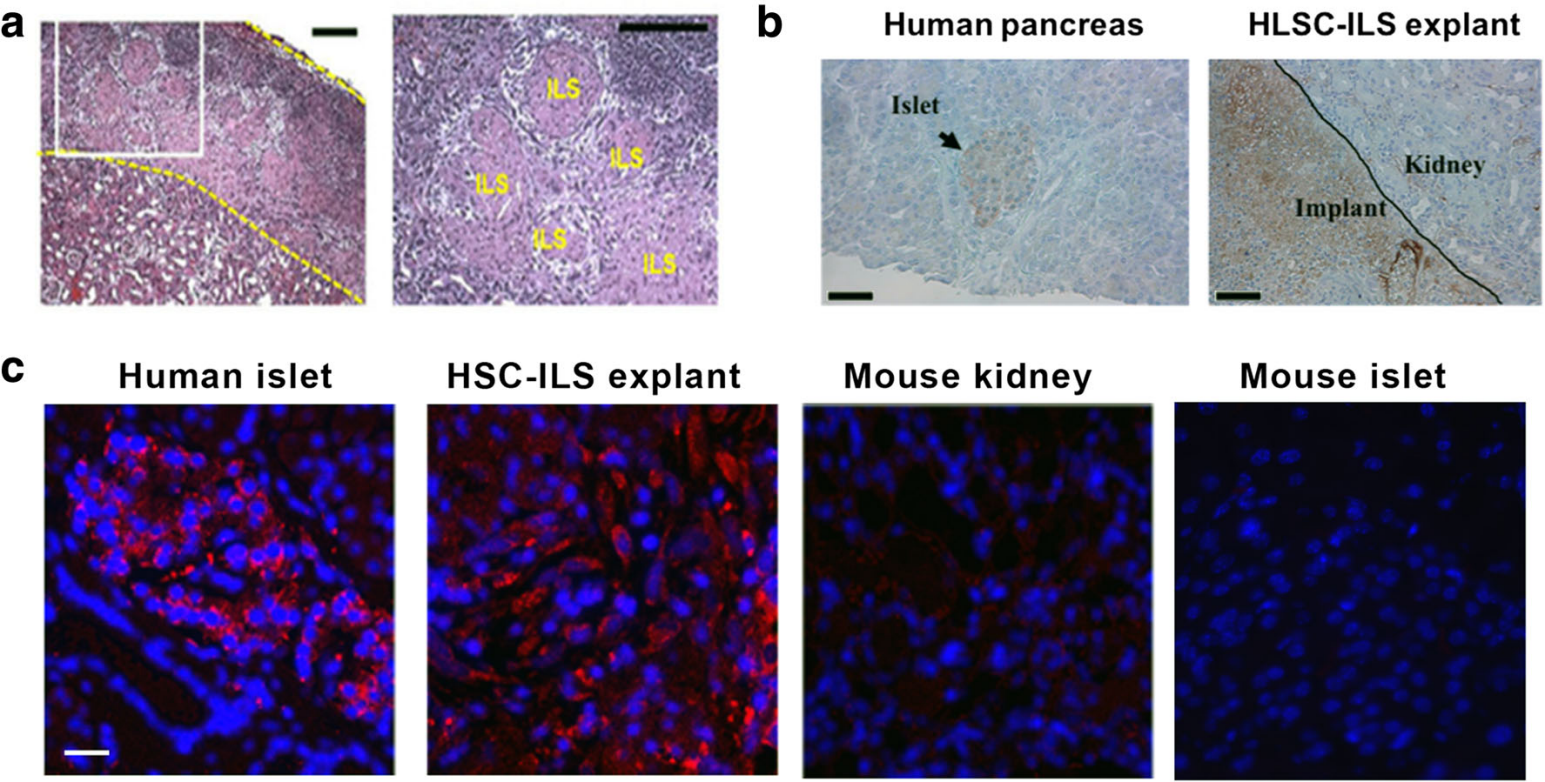
Histological characterization of HLSC-ILS explants. **a** Representative H&E micrographs showing the presence of spheroid structures under the mice renal capsule (ILS). Scale bars in micrographs are 200 μm. **b** Human insulin expression in human pancreatic tissue (left, positive control) and HLSC-ILS explants (right). Arrow is showing a human pancreatic islet while positive reactivity is showed in brown. Scale bars in micrographs are 200 μm. **c** Insulin FISH reaction (red) in human pancreatic tissue (positive control) and HLSC-ILS explants. Renal and pancreatic mouse tissue were used as negative controls. Scale bars in micrographs are 10 μm. Three experiments were done with similar results

The histological analysis demonstrated the presence of spheroid structures under the renal capsule in the right kidney of diabetic mice treated with HLSC-ILS (Fig. 9a). The expression of both protein and mRNA of human insulin in the implant was detected by immunohistochemistry and by in situ hybridization. Human islets were used as positive control and mouse kidney and pancreas as negative controls (Fig. 9b, c).

### Gene Expression Profile Shows a Further In Vivo Maturation of HLSC-ILS

**Fig. 10:**
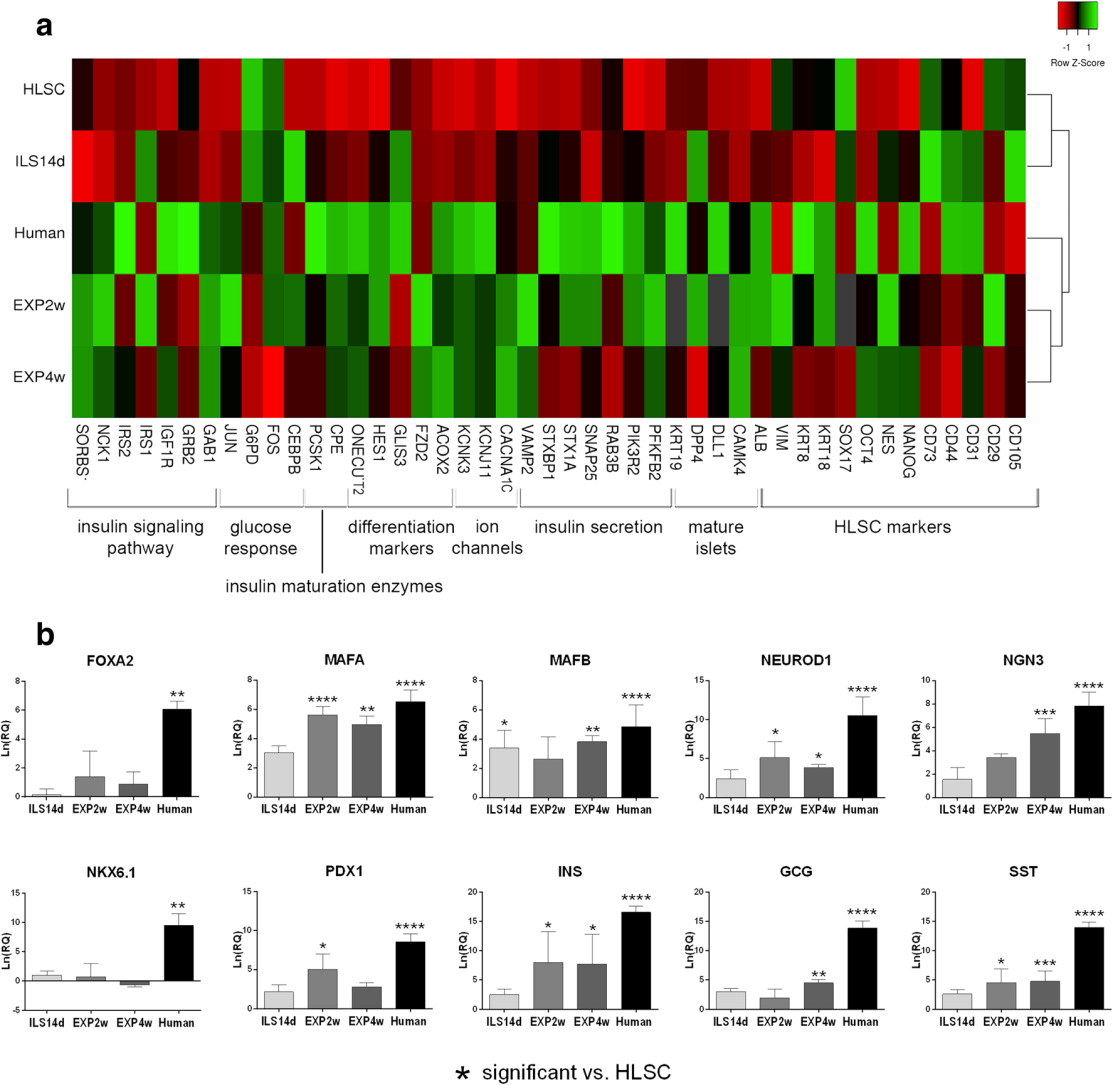
qRT-PCR array gene expression analysis of non-differentiated HLSC, HLCS-ILS before and after implantation in diabetic mice, and human islets. **a** Hierarchical clustering analysis of HLSC and HLSC-ILS at 14 days of culture in vitro (ILS 14d), HLSC-ILS explanted after 2 (EXP2w) or 4 (EXP4w) weeks of implantation in diabetic mice and human islets (Human). The analyzed genes are listed below the heat map and grouped by gene function. The expression levels are reported as row Z score of Ln(RQ) values (scale color: *red* low expression, *green* high expression) of three independent experiments run in triplicate. Average linkage clustering method and Euclidean distance measurement methods were used. The heat map was generated with heatmapper online software (http://www.heatmapper.ca/expression/). **b** Real Time PCR analysis of transcription factors involved in β cells maturation and pancreatic hormones. Values are reported as mean ± SD of Ln(RQ) of three independent experiments run in triplicate. Expression is normalized for HLSC (Ln(RQ) = 0, not shown). * *p* < 0.05, ** *p* < 0.01, *** *p* < 0.001, **** *p* < 0.0001

Comparative gene expression analysis was performed by qRT-PCR microarrays to evaluate the differentiation of HLSC-ILS after in vivo implantation (Fig. 10). The gene expression profile of HLSC, HLSC-ILS at 14 days of differentiation (ILS14d), HLSC-ILS explanted after 2 weeks (EXP2w) or 4 weeks (EXP4w) of implantation, and human islets were compared and clustered (Fig. 10a). As showed in the heat map, the expression level of the most genes was lower in HLSC and HLSC-ILS (red spots) and was higher in explanted HLSC-ILS and human islets (green spots). Moreover, ILS14d and HLSC showed a similar gene expression profile and clustered together, while EXP2w and EXP4w showed a gene expression profile closer to that of human islets. qRT-PCR showed that the expression of several transcription factors involved in β-cell differentiation (MAFA, MAFB, NEUROD1, NGN3, PDX1) and of insulin, somatostatin and glucagon was further enhanced after in vivo implantation, in EXP2w and EXP4w, compared to HLSC-ILS at 14 days of differentiation, with a statistically significant increase compared to non-differentiated HLSC (Fig. 10b).

## Discussion

In the present study, we generated islet-like structures expressing insulin/C-peptide by a one-step protocol based on protamine-dependent aggregation of HLSC. HLSC-ILS produced C-peptide after in vitro stimulation with high glucose and rapidly reversed hyperglycemia in diabetic mice.

One of the aims of cell-based therapy in diabetes is to generate new islet-like structures morphologically and functionally similar to human pancreatic islets and that can sense glucose and secrete insulin in response. A potential strategy is to promote cell aggregation into spheres [55] in the attempt to reproduce more closely the in vivo structure facilitating cell-to-cell and cell-to-matrix interactions [56].

It has been shown that the cell aggregation in 3D structures contribute to cell fate determination [57]. In contrast to cells differentiated in adherent conditions, 3D developed structures show uniform and high local cellular density, which may exert a positive influence on the uniformity of pancreatic differentiation and may potentiate the effective cell-association [57] by an E-cadherin mediated mechanism [58]. Cell aggregation into uniform clusters may favor the generation of islet-like structures containing endocrine cells.

It has been reported that the in vitro generated hESC aggregates contained mostly immature polyhormonal cells [57]. Interestingly, a complete maturation occurred only after their implantation in vivo. The pancreatic neo-grafts contained cells expressing glucagon, somatostatin, ghrelin and pancreatic polypeptide beside insulin. These cells initiated to secrete human C-peptide (5–6 weeks post-implant) and exhibited a functional response to glucose after the implant. A significant glucose-stimulated C-peptide secretion and restoration of glycemia were observed after 11–15 weeks post-implantation in diabetic mice [57, 58].

Recently, a new protocol of efficient conversion of hESC into insulin-producing cells with the capability to promote a rapid reversal (2–6 weeks) of murine diabetes has been described [20]. However, compared with cadaveric human islets, hESC derived insulin-producing cells are not fully equivalent to mature β cells because they exhibit reduced secretory properties in vitro [20]. Pagliuca et al. demonstrated the generation of stem cell-derived β cells able to secrete insulin in the serum of mice in a glucose-regulated manner after transplantation. However, most of these cells were reported to be biologically immature in vitro and acquired a complete maturation only after in vivo implantation [19].

In the pancreas α, β, γ, ε and PP cells are scattered throughout the human islet. The distinctive cytoarchitecture of islets may favor interactions between the different types of cells with consequent cell maturation [24]. This hypothesis is supported by the study of Rodríguez-Díaz et al. demonstrating that the presence of α cells provide paracrine cholinergic input for a fine tuning of normal β cell function, that primes the β cell for an optimal response to glucose [25].

Here, we demonstrated that protamine-induced HLSC aggregation stimulated spontaneous differentiation into islet-like structures depending on protamine concentration and cell-density.

Several studies have shown that hepatocytes are able to generate insulin producing cells after the induction of ectopic PDX1 gene expression with consequent inhibition of liver-specific genes [43–46]. Moreover, PDX1-induced transdifferentiation was enhanced by exendin 4, which stimulated cell proliferation and maturation [47].

HLSC is a cell population with an immature phenotype characterized by the expression of several stem cell and embryonic markers with multiple differentiation capabilities [48, 49]. In respect to human hepatocytes, which have a very low grade of in vitro proliferation, HLSC show high proliferation with a doubling time ranging from 30 to 35 hours, depending on the passage [48]. Herein, we show that HLSC have an intrinsic ability to generate insulin secreting 3D islet-like structures, possibly related to the common embryonic origin of both liver and pancreas [41, 42]. Most of the studies that aim to generate β-like cells are based on reprogramming pluripotent stem cells or adult pancreatic or non-pancreatic cells types by gene transfection or by inducing epigenetic changes [4]. Charge-dependent aggregation of HLSC in 3D spheroidal structures was per se sufficient to activate a differentiation program into insulin-producing cells. In fact, insulin production was observed at transcriptional and protein levels both in vitro and in vivo. Moreover, HLSC-ILS could secrete C-peptide in response to high glucose stimulation in both static and dynamic conditions. Gene expression analysis confirmed that HLSC-ILS cultured in vitro undergo a differentiation process, which is further enhanced after in vivo implantation. HLSC-ILS co-expressed PDX1 and NGN3. The latter genes typical of pancreatic progenitors drive the secondary transition from pancreatic progenitors to endocrine progenitors [59]. NGN3 expression is restricted to immature cells and levels and timing of its expression are critical for the specific differentiation into β-cells [59]. In mature β-cells, PDX1 regulates β-cell maintenance [60] and insulin transcription [61]. The overexpression of NANOG and nestin by HLSC-ILS suggest an early stage differentiation phenotype. Nestin, in particular, was shown to play a role in stem cell differentiation into insulin-producing cells [62]. SOX17, and FOXA2 regulate the development of pancreatic endoderm in the primary transition of embryonal pancreas development [63]. These genes were increased in HLSC-ILS at 4 days of differentiation, but not at 7 and 14 days, suggesting an early differentiation phase at day 4. Moreover, HES1 and DLL1, which take part in Notch signaling in early pancreatic development [64], were increased in HLSC-ILS. GLIS3, which contribute to NGN3 expression in embryo [65] and to insulin transcription in β-cells [66] was also increased in HLSC-ILS. Other transcription factors involved in later phases of pancreatic development were upregulated in HLSC-ILS compared to HLSC: NKX6.1 drives the differentiation of NGN3 positive pancreatic progenitor towards the endocrine lineage and then into β-cells [67, 68], MAFA and MAFB are involved in β-cells maturation and final differentiation [69, 70], and NEUROD1 promotes β-cell differentiation and insulin transcription [59]. These data suggest that HLSC-ILS may be composed by cells in several stages of development from stem cells to mature pancreatic β-cells. Moreover, several transcripts encoding for proteins involved in docking and fusion of insulin granules with the plasma membrane (PFKFB2, STX1A, STXBP1, VAMP2) [71–73] were overexpressed in HLSC-ILS with respect to HLSC. Similarly, voltage gated calcium (CACNA1C) [74] and potassium (KCNJ11) [75] channels, which trigger insulin secretion were expressed after differentiation. These results showed that insulin secretion machinery was activated in HLSC-ILS compared to HLSC, especially at 7 and 14 days of differentiation. Notably, CPE and PCSK1, two enzymes involved in proinsulin maturation and cleavage of C-peptide [76, 77] were significantly upregulated in HLSC-ILS at day 14, suggesting their ability to produce insulin, as confirmed by the secretory assay. Overall, we can conclude that in vitro HLSC-ILS display an immature phenotype with a progressive differentiation during prolonged culture.

As seen for hESC [20], HLSC-ILS in basal conditions produced significantly lower C-peptide when compared with cadaveric human islets. However, HLSC-ILS were shown to increase C-peptide production in response to high glucose concentrations in vitro in both static and dynamic conditions. In dynamic conditions the shift from HG to LG was followed by a reduction of C-peptide secretion. We also observed that after in vivo implantation, HLSC-ILS were able to significantly reduce hyperglycemia in streptozotocin-diabetic SCID mice after 1 week of implantation and to restore a normal profile of glucose tolerance test. Reduction of hyperglycemia was associated with detection of human C-peptide in diabetic mice, which in turn exhibited very low, and in some animal undetectable, levels of murine C-peptide. As suggested by Kroon et al. [35] the observation of a latency in reduction of glycemia may depend on a further in vivo maturation of transplanted islet-like structures and/or on the need of a longer period of time for the engraftment, as described for human islet transplantation [3, 35]. Removal of the HLSC-ISL implants resulted in an increase of blood glucose levels and a return to diabetic profile following IPGTT, suggesting that the implanted HLSC-ILS were responsible for reversal of diabetes in the recipient mice.

An in vivo further maturation of HLSC-ILS was also supported by comparative gene expression analysis among HLSC, in vitro differentiated HLSC-ILS, explanted HLSC-ILS, and human islets. The expression of several genes involved in insulin signaling pathway, glucose response, differentiation, ion channels, insulin secretion and mature islets were upregulated in explants compared to the in vitro differentiated HLSC-ILS and HLSC. As shown in the heat map (Fig. 10a), the gene expression profile of explanted HLSC-ILS was closely associated to human islets than to HLSC ILS cultured in vitro and HLSC, suggesting a further in vivo maturation. Interestingly, two markers of immature β-cells, Acox2 and Fzd2 [54], were overexpressed in EXP-ILS compared to both HLSC-ILS and human islets, which were respectively less and more differentiated. Notably, three genes for adaptor proteins involved in the transduction of insulin signal (GAB1, NCK1, SORBS1) [78, 79] were expressed at similar or higher levels in EXP-ILS compared to human islets, and were upregulated in respect to HLSC-ILS and HLSC, suggesting an increased activation of the insulin signaling pathway. Similarly, several markers of insulin secretion (CACNA1C, KCNJ11, KCNK3, PFKFB2, PIK3R2, SNAP25, VAMP2) [71, 80–82] were expressed at similar or higher levels in EXP-ILS and human islets and were expressed at lower levels in HLSC and HLSC-ILS, suggesting an increased ability of EXP-ILS to release insulin granules.

In conclusion, we describe a one-step simple and reproducible protocol for scalable production of islet-like structures that are capable to restore normoglycemia in a pre-clinical model of diabetes. Despite the demonstration that multiple stem cell types are able to generate insulin-producing cells, the protocols remain complex and often require cell transfection [83–85]. HLSC have an intrinsic ability to undergo endocrine differentiation when aggregated in 3D spheroid structures. HLSC is a cellular source that can be easily obtained and expanded from a small liver biopsy of adult subjects [48]. Pre-clinical studies have shown the biosafety of these cells, which have been recognized by the European Medical Agency as orphan drug for liver disorders (EU/3/12/971; EU/3/11/904; EU/3/12/983). Although the in vitro differentiated HLSC-ILS are relatively immature and not fully comparable to human mature β-cells, they further differentiate in vivo and reverse hyperglycemia in diabetic mice. Further studies are needed to improve the in vitro differentiation of HLSC, to obtain more mature islet-like structures which may accelerate diabetes reversal, and to investigate the possibility to use HLSC-ILS as a candidate for a cell-based therapy for diabetes.

## Electronic supplementary material

ESM 1 (PNG 1162 kb)

High Resolution Image (TIF 1969 kb)

ESM 2 (DOCX 36 kb)
