# Supplementary figures and images for: Islet-Like Structures Generated In Vitro from Adult Human Liver Stem Cells Revert Hyperglycemia in Diabetic SCID Mice
Source: Stem Cell Rev. 2018 Sep 6;15(1):93–111. doi: 10.1007/s12015-018-9845-6 (PMC6510809; doi:10.1007/s12015-018-9845-6)

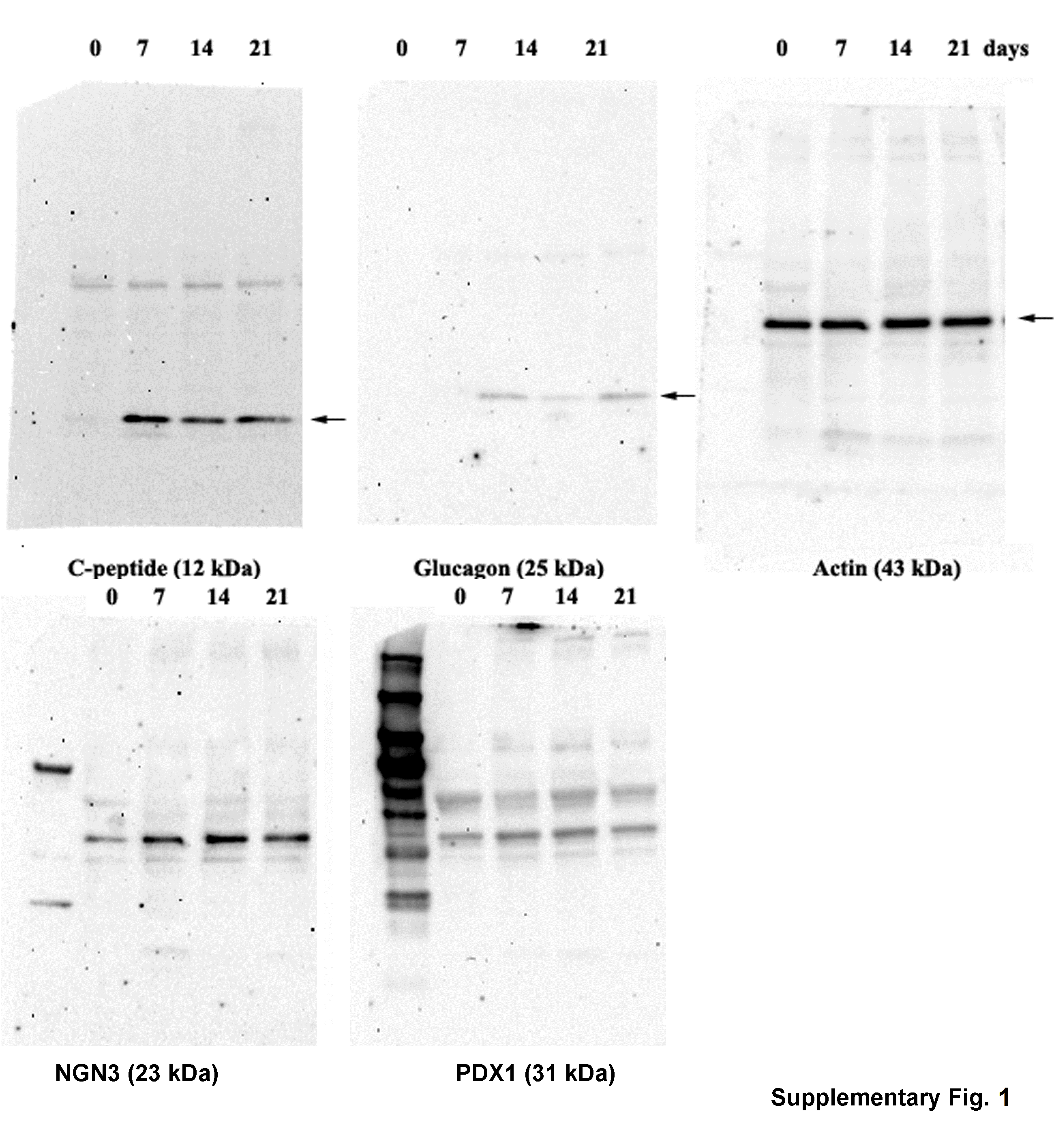

Supplement: Supplementary file 1 — (PNG 1162 kb) [file 12015_2018_9845_Fig11_ESM.png]

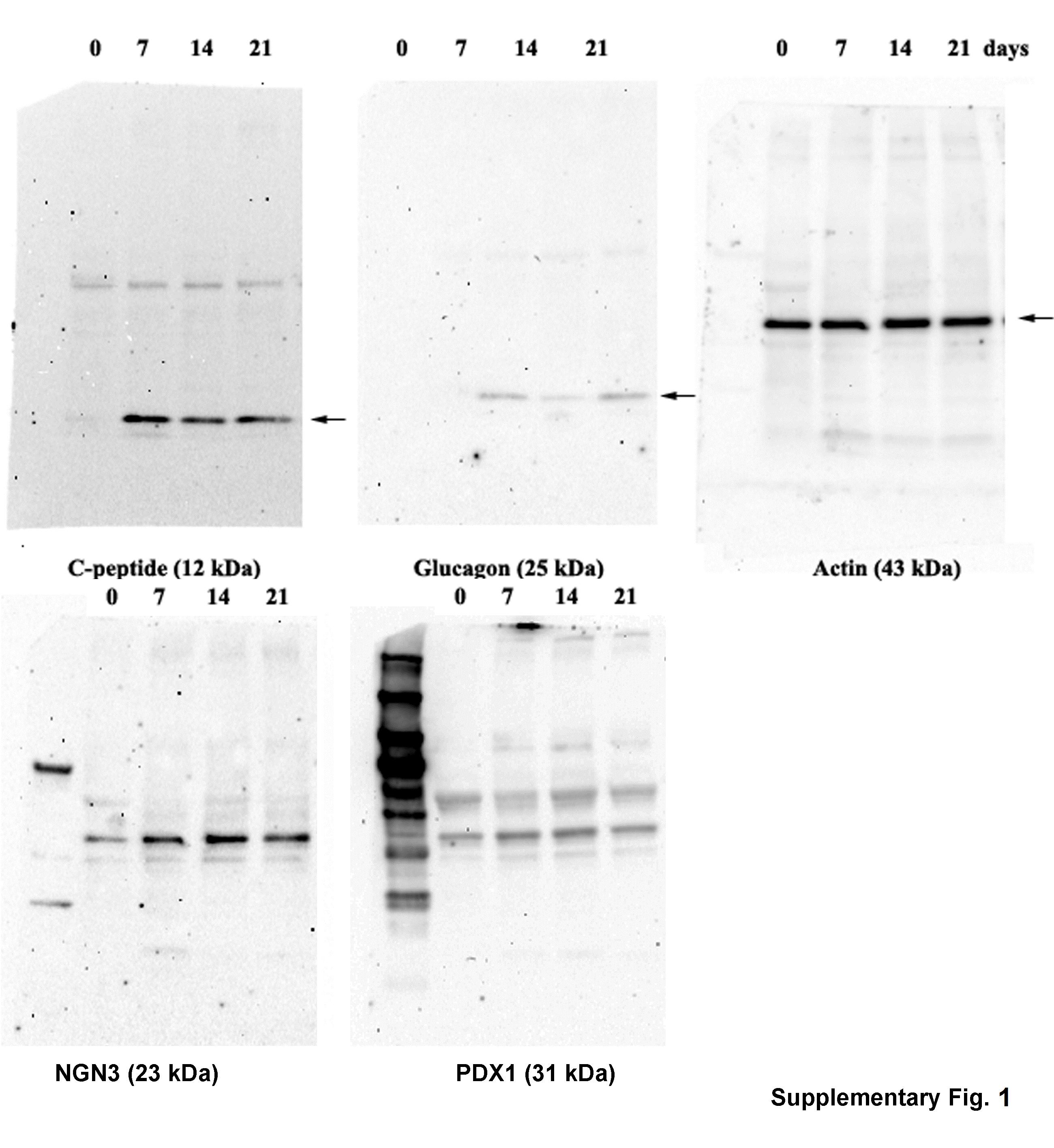

Supplement: Supplementary file 2 — High Resolution Image (TIF 1969 kb) [file 12015_2018_9845_MOESM1_ESM.tif]
