# Supplementary material for: Islet-Like Structures Generated In Vitro from Adult Human Liver Stem Cells Revert Hyperglycemia in Diabetic SCID Mice
Source: Stem Cell Rev. 2018 Sep 6;15(1):93–111. doi: 10.1007/s12015-018-9845-6 (PMC6510809; doi:10.1007/s12015-018-9845-6)
Supplement: Supplementary file 3 — (DOCX 36 kb) [file 12015_2018_9845_MOESM2_ESM.docx]

Supplementary Table I:

List of target gene spotted on TaqMan array microfluidic card:

| **Assay ID** | **Gene Symbol(s)** | **Gene Name(s)** |
| --- | --- | --- |
| Hs99999907_m1 | B2M | beta-2-microglobulin |
| Hs99999908_m1 | GUSB | glucuronidase beta |
| Hs99999909_m1 | HPRT1 | hypoxanthine phosphoribosyltransferase 1 |
| Hs00185873_m1 | ACOX2 | acyl-CoA oxidase 2 |
| Hs00609411_m1 | ALB | Albumin |
| Hs00167681_m1 | CACNA1C | calcium voltage-gated channel subunit alpha1 C |
| Hs00174318_m1 | CAMK4 | calcium/calmodulin dependent protein kinase IV |
| Hs01075864_m1 | CD44 | CD44 molecule (Indian blood group) |
| Hs00942496_s1 | CEBPB | CCAAT/enhancer binding protein beta |
| Hs00175676_m1 | CPE | carboxypeptidase E |
| Hs00194509_m1 | DLL1 | delta like canonical Notch ligand 1 |
| Hs00897391_m1 | DPP4 | dipeptidyl peptidase 4 |
| Hs00923996_m1 | ENG | Endoglin |
| Hs04194186_s1 | FOS | Fos proto-oncogene, AP-1 transcription factor subunit |
| Hs00361432_s1 | FZD2 | frizzled class receptor 2 |
| Hs00166169_m1 | G6PD | glucose-6-phosphate dehydrogenase |
| Hs00157646_m1 | GAB1 | GRB2 associated binding protein 1 |
| Hs00541450_m1 | GLIS3 | GLIS family zinc finger 3 |
| Hs00157817_m1 | GRB2 | growth factor receptor bound protein 2 |
| Hs00172878_m1 | HES1 | hes family bHLH transcription factor 1 |
| Hs00609566_m1 | IGF1R | insulin like growth factor 1 receptor |
| Hs00178563_m1 | IRS1 | insulin receptor substrate 1 |
| Hs00275843_s1 | IRS2 | insulin receptor substrate 2 |
| Hs01127536_m1 | ITGB1 | integrin subunit beta 1 |
| Hs99999141_s1 | JUN | Jun proto-oncogene, AP-1 transcription factor subunit |
| Hs00265026_s1 | KCNJ11 | potassium voltage-gated channel subfamily J member 11 |
| Hs00605529_m1 | KCNK3 | potassium two pore domain channel subfamily K member 3 |
| Hs02827483_g1 | KRT18 | keratin 18 |
| Hs00761767_s1 | KRT19 | keratin 19 |
| Hs01595539_g1 | KRT8 | keratin 8 |
| Hs02387400_g1 | NANOG | Nanog homeobox |
| Hs01592377_m1 | NCK1 | NCK adaptor protein 1 |
| Hs00707120_s1 | NES | Nestin |
| Hs00159686_m1 | NT5E | 5'-nucleotidase ecto |
| Hs00191477_m1 | ONECUT2 | one cut homeobox 2 |
| Hs01026107_m1 | PCSK1 | proprotein convertase subtilisin/kexin type 1 |
| Hs01065279_m1 | PECAM1 | platelet and endothelial cell adhesion molecule 1 |
| Hs01015408_m1 | PFKFB2 | 6-phosphofructo-2-kinase/fructose-2,6-biphosphatase 2 |
| Hs00178181_m1 | PIK3R2 | phosphoinositide-3-kinase regulatory subunit 2 |
| Hs00742896_s1 | POU5F1 | POU class 5 homeobox 1 |
| Hs01001137_m1 | RAB3B | RAB3B, member RAS oncogene family |
| Hs00938957_m1 | SNAP25 | synaptosome associated protein 25 |
| Hs00908953_m1 | SORBS1 | sorbin and SH3 domain containing 1 |
| Hs00751752_s1 | SOX17 | SRY-box 17 |
| Hs00270282_m1 | STX1A | syntaxin 1A |
| Hs01119036_m1 | STXBP1 | syntaxin binding protein 1 |
| Hs00360269_m1 | VAMP2 | vesicle associated membrane protein 2 |
| Hs00958111_m1 | VIM | Vimentin |
